# A Rho Scaffold Integrates the Secretory System with Feedback Mechanisms in Regulation of Auxin Distribution

**DOI:** 10.1371/journal.pbio.1000282

**Published:** 2010-01-19

**Authors:** Ora Hazak, Daria Bloch, Limor Poraty, Hasana Sternberg, Jing Zhang, Jiří Friml, Shaul Yalovsky

**Affiliations:** 1 Department of Plant Sciences, Tel Aviv University, Tel Aviv, Israel; 2 Department of Plant Systems Biology, Flanders Institute for Biotechnology (VIB), Ghent, Belgium; 3 Department of Plant Biotechnology and Genetics, Ghent University, Ghent, Belgium; University of York, United Kingdom

## Abstract

Development in multicellular organisms depends on the ability of individual cells to coordinate their behavior by means of small signaling molecules to form correctly patterned tissues. In plants, a unique mechanism of directional transport of the signaling molecule auxin between cells connects cell polarity and tissue patterning and thus is required for many aspects of plant development. Direction of auxin flow is determined by polar subcellular localization of PIN auxin efflux transporters. Dynamic PIN polar localization results from the constitutive endocytic cycling to and from the plasma membrane, but it is not well understood how this mechanism connects to regulators of cell polarity. The Rho family small GTPases ROPs/RACs are master regulators of cell polarity, however their role in regulating polar protein trafficking and polar auxin transport has not been established. Here, by analysis of mutants and transgenic plants, we show that the ROP interactor and polarity regulator scaffold protein ICR1 is required for recruitment of PIN proteins to the polar domains at the plasma membrane. *icr1* mutant embryos and plants display an a array of severe developmental aberrations that are caused by compromised differential auxin distribution. ICR1 functions at the plasma membrane where it is required for exocytosis but does not recycle together with PINs. ICR1 expression is quickly induced by auxin but is suppressed at the positions of stable auxin maxima in the hypophysis and later in the embryonic and mature root meristems. Our results imply that ICR1 is part of an auxin regulated positive feedback loop realized by a unique integration of auxin-dependent transcriptional regulation into ROP-mediated modulation of cell polarity. Thus, ICR1 forms an auxin-modulated link between cell polarity, exocytosis, and auxin transport-dependent tissue patterning.

## Abstract 2

In plants, auxin distribution and tissue patterning are coordinated via a feedback loop involving the auxin-regulated cell polarity factor ICR1 and the secretory machinery.

## Author Summary

The coordination of different cells during pattern formation is a fundamental process in the development of multicellular organisms. In plants, a unique mechanism of directional transport of the signaling molecule auxin between cells demonstrates the importance of cell polarity for tissue patterning. The direction of auxin flow is determined by polar subcellular localization of auxin transport proteins called PINs, which facilitate auxin efflux. At the same time, an auxin-mediated positive feedback mechanism reinforces the polar distribution of PINs. However, the molecular mechanisms that underlie polar PIN localization are not well understood. In eukaryotic cells, the Rho family of small GTPases function as central regulators of cell polarity. We show that a Rho-interacting protein from plants, called ICR1, is required for recruitment via the secretory system of PIN proteins to polar domains in the cell membrane. As a result, ICR1 is required for directional auxin transport and distribution and thereby for proper pattern formation. In addition, both the expression and subcellular localization of ICR1 are regulated by auxin, suggesting that ICR1 could function in a positive feedback loop that reinforces auxin distribution. Thus, ICR1 forms an auxin-modulated link between cell polarity, protein secretion, and auxin-dependent tissue patterning.

## Introduction

ROP (Rho of Plants), also known as RAC GTPases, have been implicated as master regulators of cell polarity [1],[2]. In their GTP-bound, active state, ROPs interact with downstream effector proteins to regulate organization of actin and microtubules (MT), vesicle trafficking, production of phosphoinositides, and gradients of reactive oxygen species (ROS) and Ca^2+^ [1]–[6]. ROPs function at the plasma membrane to which they attach by virtue of posttranslational lipid modifications prenylation and *S*-acylation [1],[7]–[9]. The ability of ROPs to interact with membranes allows these proteins to regulate actin polymerization and vesicle trafficking at discrete sites of the plasma membrane and of internal membranes, which is essential for their role in the control of cell polarity [10]. ROPs are polarly localized and expression of activated dominant ROP mutants that cannot hydrolyze GTP compromises cell polarization [1]–[6] and inhibits endocytic vesicle recycling [3]. Due to their essential role in generation of cell polarity, it was plausible that ROPs also regulate distribution of polar cargos including PIN auxin efflux transporters [11].

We have previously identified a ROP interacting coiled coil domain scaffold protein Interactor of Constitutive active ROP 1 (ICR1) and demonstrated that it affects cell polarity [12]. Recently, ICR1/RIP1 has been implicated as a regulator of polar pollen tube growth [13]. The primary root meristem of *icr1* mutant and *RNAi* silenced plants collapses soon after germination, resembling mutants affected in basal localization of PIN proteins and multiple *pin* loss-of-function mutants [14]. These results suggested that ICR1 might form a link between Rho-regulated cell polarity and polar auxin transport.

Auxin is the major signal for tissue polarity and patterning in plants. Polar auxin transport and resulting asymmetric auxin distribution within tissues (auxin maxima and gradients) are essential for proper development of the embryo, the root, and the shoot, differentiation and regeneration of vascular tissues, and for tropic responses [14]–[18]. Directionality of auxin transport depends on polar subcellular distribution of PINFORMED (PIN) family of efflux transporters [11],[19],[20]. Dynamic PIN polarity is a result of constitutive endocytic recycling. Recycling to the membrane requires the function of the brefeldin A (BFA)-sensitive ADP ribosylation factor GDP/GTP Exchange Factors (ARF GEFs) [21],[22]. The endocytic step is clathrin dependent and requires function of endosomal Rab5/Ara7 [14],[23]. However, little is known on how PIN recycling is directed to result in their polar distribution.

In this work we show that ICR1 is an essential component of the auxin transport machinery functioning in exocytosis and as a part of an auxin modulated feedback loop. Thus, ICR1 links between Rho-regulated cell polarity and auxin associated pattern formation.

## Results

### Auxin Distribution in *icr1* Roots

**Figure 1:**
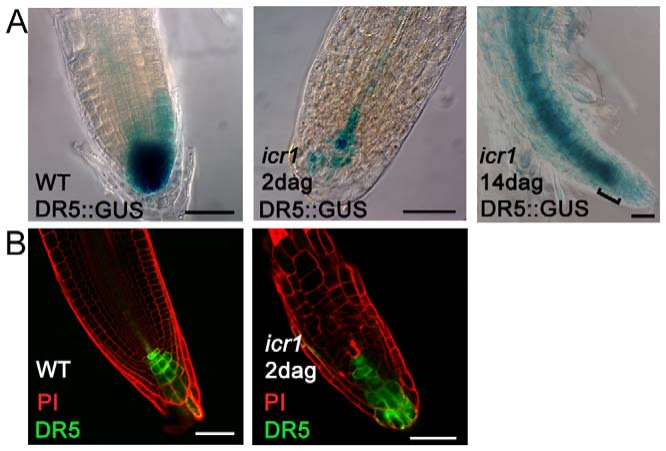
Compromised auxin distribution in *icr1* roots. (A) *DR5::*GUS in WT and 2 and 14-d-old *icr1* seedlings. (B) *DR5rev::ER-GFP* in WT and 2-d-old *icr1* seedlings. Note the displacement of the auxin maximum toward the root cup in *icr1* (B) and the accumulation of auxin signal in the stele (A). In 14-d-old plants auxin did not reach the meristem and accumulated in the stele (A brackets). Bars correspond to 50 µm.

To address the potential function of ICR1 in auxin transport, we examined auxin distribution in wild type (WT) *Col-0* and *icr1* mutant plants using the auxin sensitive reporters *DR5::GUS* and *DR5rev::GFP* [17],[18]. The formation of the *DR5*-visualized auxin maximum in the quiescent center (QC) and proximal columella cells is required for root meristem maintenance and correct tissue patterning [18]. In young 2 days after germination (DAG) *icr1* roots the auxin maximum was displaced towards the distal tier of root cap and was reduced (Figure 1A and B). Concomitant with the reduction of the auxin maximum at the root tip, it started to accumulate in the vascular bundle (Figure 1A). In older (14 DAG) roots auxin accumulated in the vascular bundle but did not reach the tip (Figure 1A). These results suggested that the collapse of the root meristem in *icr1* plants resulted from compromised auxin transport and in-turn gradual disappearance of the auxin maximum at the root tip.

### Columella Specification in *icr1*

**Figure 2:**
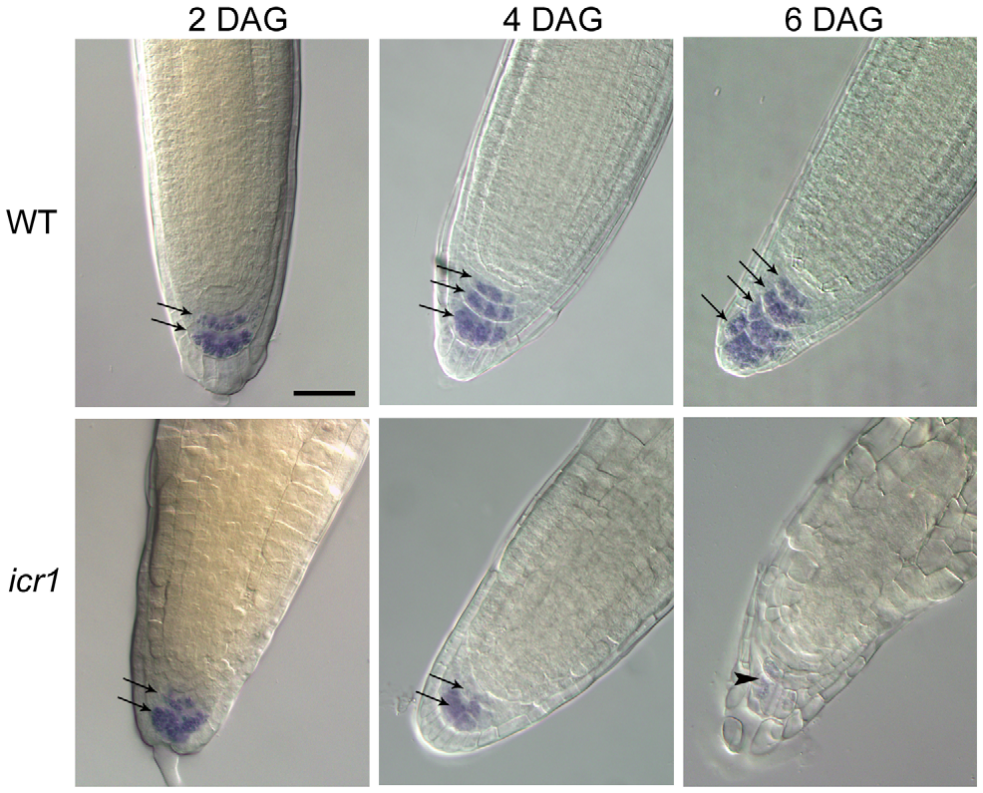
Columella specification in WT and *icr1* roots. Lugol's (IKI) staining was used for detection of starch granules in 2, 4, and 6 DAG WT and *icr1* seedlings. In WT roots, the number of columella tiers containing starch granules increase with the age of seedlings (arrows). In *icr1* roots, at 2 DAG, two tiers of cells containing starch granules could be detected (arrows), whereas at older stages starch granules staining decreased and almost completely disappears at 6 DAG (arrowhead). The bar corresponds to 50 µM for all images.

The specification of the columella at the root tip is closely associated with the formation of a stable auxin maximum [18]. Columella cells contain starch granules that can be easily identified by staining of roots with Lugol staining (IKI). No starch granules were detected in the collapsed primary roots of *icr1* [12], indicating that columella identity was lost. Because the stable auxin maximum was displaced in *icr1* embryos and roots, it was plausible that columella cells may be specified in young roots but would later disappear, concomitant with the proximal shift of the auxin maximum. To examine the specification of the columella cells, the existence of starch granules was examined in WT and *icr1* roots at 2, 4, and 6 DAG (Figure 2). In 2 DAG seedlings, starch granules were detected in two cell tiers, in both WT and *icr1* root tips. At 4 DAG, starch granules were detected in 3 cell tiers of WT plants but remained confined to 2 cell tiers in the *icr1* roots. Furthermore, the columella initials could not be detected in *icr1*. At 6 DAG, WT roots had 4 columella cell tiers, while starch granules were barely detected or absent in the *icr1* roots (Figure 2). These results indicate that columella identity is initially specified in *icr1* root tip cells and then gradually disappears, concomitant with the diminishing local auxin maximum.

### Development of *icr1* Embryos

**Figure 3:**
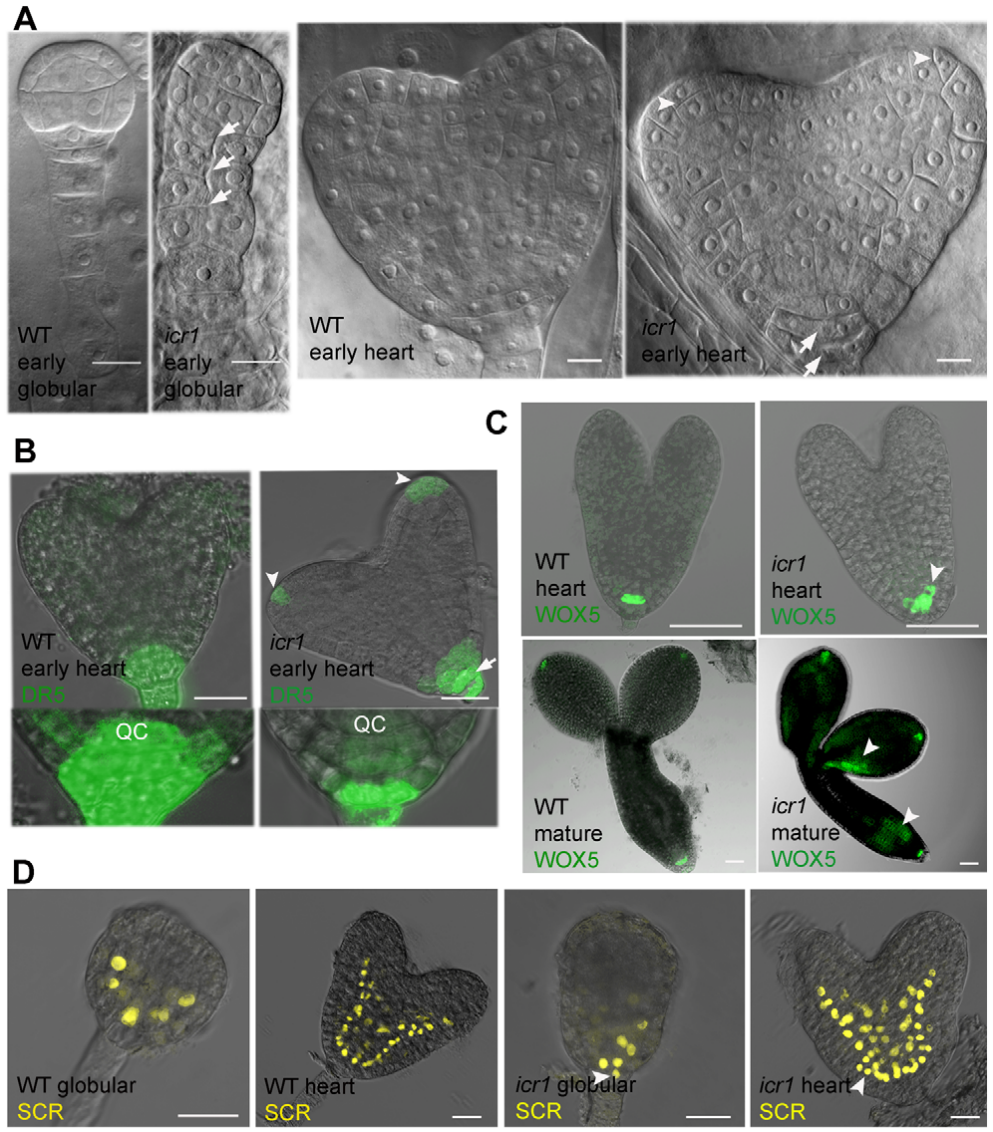
Patterning defects in *icr1* embryos are associated with altered distribution of auxin. (A) Early and late patterning defects in *icr1*, as revealed by Nomarsky DIC of cleared embryos. Approximately 10% of *icr1* embryos showed early developmental defects at the globular stage. Arrows denote abnormal cell divisions in the suspensor and hypophysis. The majority (90%) of the embryos developed normally up to the triangular stage. At the heart stage, abnormal cell divisions in the embryonic root meristem are seen (arrows). Note the non-steriotipic divisions of the protoderm in the cotyledons (arrowheads). (B) Auxin distribution in *icr1* embryos revealed by the *DR5rev*::ER-GFP marker. Arrow indicates shift in auxin maxima toward the low columella layers. Arrowheads point to the strong auxin signals at the tip of the cotyledons. Insets are single confocal scans throughout the middle of the embryonic root meristem showing the reduced auxin accumulation in QC and upper columella layers. (C) Expression of the QC marker *pWOX5::*ER-GFP. Arrowheads denote altered accumulation in heart and mature *icr1* embryos. (D) Expression of the endodermis marker *pSCR*::YFP-H2b. Arrowheads indicate spreading of the marker to adjacent cell layers in *icr1*. Bars correspond to 10 µm (A), 20 µm (B), and 50 µm (C and D). For additional information and high resolution images, see Figures S1, S2, S3, S4, S5, S6.

In plants, the polar shoot to root axis and the primary shoot and root meristems develop during embryogenesis. These developmental processes are associated with stereotypical series of cell divisions and gene expression and depend on auxin distribution [17]. Development of WT and *icr1* mutant embryos was analyzed to further establish the role of ICR1 in auxin distribution and embryo development. About 10% (27/273) of the *icr1* embryos showed defects in stereotypic cell divisions of the basal embryo pole at the globular stage as well as abnormal divisions in the suspensor, including the uppermost cell, which forms hypophysis (Figure 3A and Figure S1). The majority (90%, 246/273) of the mutant embryos developed normally through the globular stage (Figure S2 and Table S1). From the triangular stage and onward, abnormal divisions of the suspensor, QC and columella cells were detected. Moreover, abnormal division planes were observed in the protoderm at the position of future cotyledons (Figure 3A, Figure S2, and Table S1). The variable penetrance of the *icr1* mutation also resulted in reduced developmental synchronization of embryos within single siliques. Whereas in WT siliques the embryos were found in two to three developmental stages, in *icr1* siliques the embryos were spread between four and five stages (Figure S3), indicating that developmental delay in *icr1* occurs at different stages. The data suggested that the loss of *ICR1* function results in a gradient of phenotypic defects primarily at the places of auxin maxima and in processes requiring differential auxin distribution.

### Auxin Distribution in *icr1* Embryos

Next we examined the auxin distribution in embryos to determine its association with the *icr1* phenotype (Figure 3B and Figure S4). In comparison to WT, in the *icr1* embryos the auxin response maximum was shifted distally from the QC to the lower tier of the future columella cells (Figure 3B, arrow). Furthermore, ectopically strong *DR5* activity was detected at the tip of the cotyledons (Figure 3B, arrowheads, and Figure S4). The abnormal auxin distribution coincided with the non-stereotypic divisions of the future root meristem, further suggesting that the developmental abnormalities in *icr1* result from compromised auxin distribution.

### Patterning of *icr1* Embryos and Roots

Root and embryo patterning and QC maintenance depend on highly specific expression pattern and subcellular localization of transcriptional regulators, including WOX5 (WUSCHEL related homeobox 5) and SCR (SCARECRAW), that define the stem cell niche [24]–[26]. In roots, formation of a stable auxin maximum is required for correct expression pattern of *WOX5*, *SCR*, and an additional marker SHORTROOT (SHR) [18],[27]. To verify that ICR1 is indeed involved in regulation of auxin distribution rather than this gene network, the expression of *WOX5* and *SCR* in embryos was examined (Figure 3C and 3D and Figures S5 and S6). Both *WOX5* and *SCR* were expressed in the *icr1* mutant embryos, but their expression pattern was altered, reflecting changes in cell identity and disruption of the embryo polar axis. The embryo development and auxin response further suggested that the alterations in *icr1* mutant plants are related to defects in auxin distribution.

**Figure 4:**
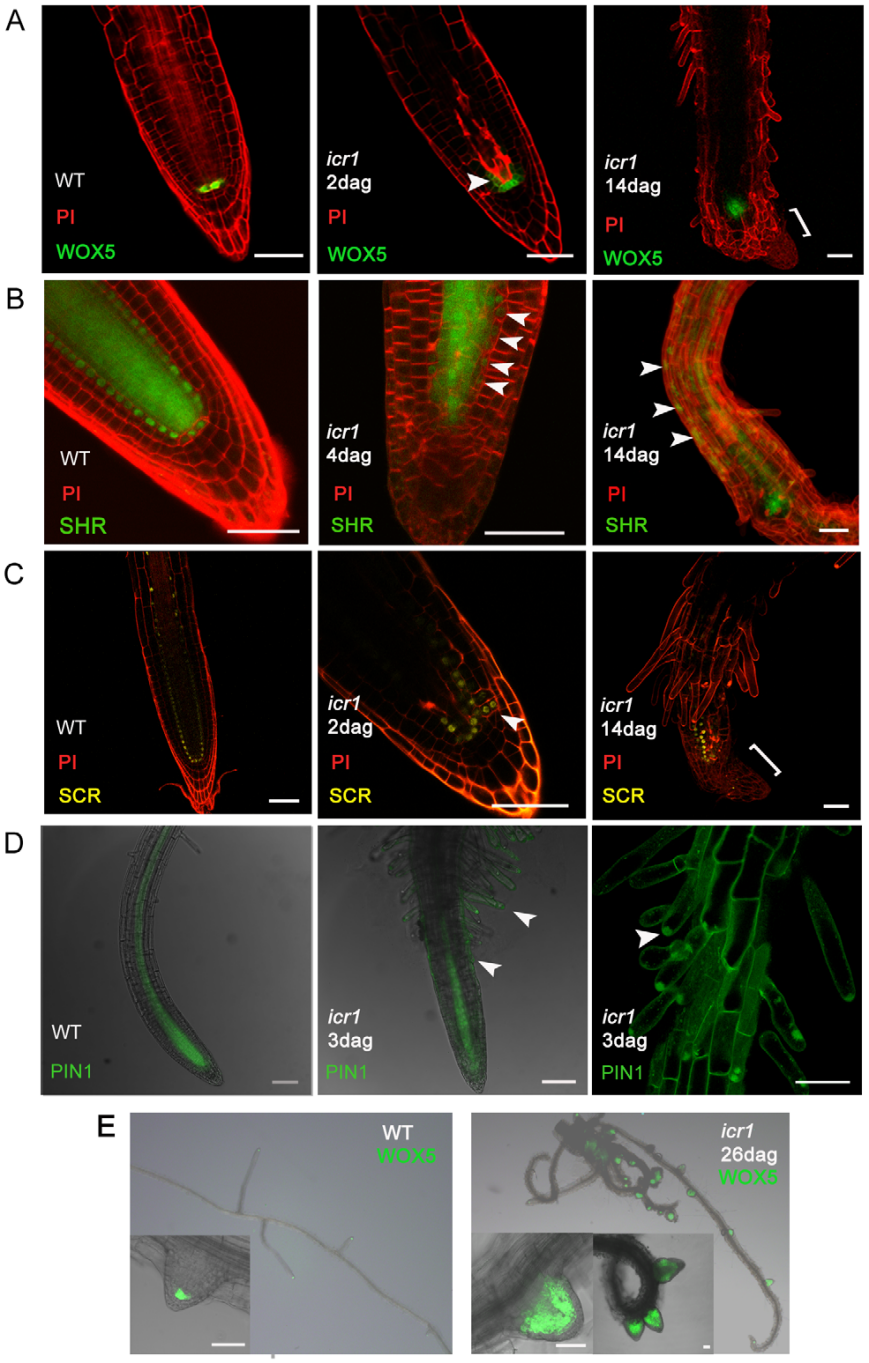
Abnormal expression of patterning markers in *icr1* roots. In *icr1* roots, expression of the QC and stem cells markers *pWOX5::*ER-GFP (A), *pSHR::*YFP-SHR (B), and *pSCR::*YFP-H2b (C) spread to the neighboring cells in 2- to 4-d-old seedlings and was diminished in old (14-d-old) roots meristems and coincidently appeared at more apical locations at the sites of auxin accumulation (brackets). Note the expression and cytoplasmic localization of SHR in epidermis (arrowheads). (D) Expression pattern of *pPIN1*::GFP-PIN1 in WT and *icr1* roots. Note the abnormal expression pattern of GFP-PIN1 in the epidermis and root hairs in *icr1* roots (arrowheads). (E) Irregular spacing (right inset) and arrested growth of lateral roots soon after emergence seen in an *icr1* mutant plant. Note the abnormal expression pattern of *WOX5* in the *icr1* lateral root primordia (insets). Bars correspond to 50 µm.

To further establish that the altered patterning in *icr1* mutants resulted from compromised auxin transport rather than the genetic framework that regulates root development, we compared the expression of *WOX5*, *SCR*, and SHR in WT and *icr1* roots. This analysis showed that like in embryos, all the three markers were expressed in the *icr1* roots (Figure 4). Similar to laser ablation experiments of the root meristem [27], expression of *WOX5*, *SCR*, and SHR appeared at more proximal locations in older, 14-d-old, *icr1* roots and was associated with a formation of a new auxin maximum (Figure 4A–C and Figure 1). The abnormal expression pattern of *WOX5*, *SCR*, and SHR in roots further suggested that the compromised root development of *icr1* is associated with altered auxin distribution.

To further examine the function of ICR1 in root patterning, we examined the expression pattern of PIN1 in *icr1* roots. In the root, PIN1 is expressed mainly in the vascular cylinder. In *icr1* mutant roots, however, *pPIN1*-driven PIN1-GFP expression was detected also in the epidermis and root hairs where it formed intracellular aggregates (Figure 4D). The mis-expression of PIN1 was a further indication that the altered auxin distribution in *icr1* mutant plants resulted from compromised auxin transport and is not an indirect effect of perturbations in auxin-independent patterning mechanisms.

### Lateral Root Development in *icr1*

The initiation and growth of lateral roots are separable events that depend on local auxin accumulation and polar transport [15],[28]. Given the function of ICR1 in primary root development, it was plausible that it would affect development of lateral roots. The primary root of *icr1* mutant plants collapses before formation of lateral roots. However, the mutant plants develop adventitious roots that grow for some time and then collapse [12]. The formation of these adventitious roots is preceded by formation of local auxin maxima (unpublished data). Lateral roots were initiated on these adventitious roots, but their growth was arrested after emergence (Figure 4E). Often, multiple initiations of lateral roots were observed (Figure 4E, inset), indicating that the onset as well as growth of lateral roots was perturbed in *icr1*. In the *icr1* arrested lateral roots, *WOX5* was mis-expressed (Figure 4E, compare left and right panels), indicating that the new root meristem was not properly setup. Taken together, these results indicate that in *icr1* mutant plants the basic genetic framework that regulates root development is present and that the root meristem collapse, the altered organ development, and changes in cell identities can be attributed primarily to the compromised auxin distribution.

The function of roots as an auxin sink has long been postulated to play a major role in vascular differentiation [29]. The “canalization hypothesis,” which became a hallmark for explaining auxin-dependent patterning, postulates that differentiation and regeneration of vascular tissues depend on an auxin-regulated positive feedback loop [14],[16],[30]. That is, auxin induces a process that enhances its own transport from cells while inhibiting its transport in neighboring cells. This process eventually leads to the formation of auxin transporting cell files that differentiate into vascular tissues. Given the involvement of ICR1 in auxin transport, we suspected that the vascular tissues in *icr1* mutant plants would be abnormal. Transverse sections through rosette leaves were prepared to compare the vascular tissues in WT and *icr* mutant plants. Analysis of these sections showed that the leaf veins in *icr1* mutant plants are much reduced compared to WT (Figure S7). The reduced vascular tissues in *icr1* mutants further suggested the involvement of ICR1 protein in auxin transport and that it could be part of an auxin modulated feedback loop.

In summary, the detailed phenotype analyses using markers for the auxin response and major regulators of patterning in multiple auxin-regulated processes revealed pronounced defects in *icr1* mutant plants that presumably result from the defects in auxin distribution.

### Localization of PIN1 and PIN2 in *icr1*

**Figure 5:**
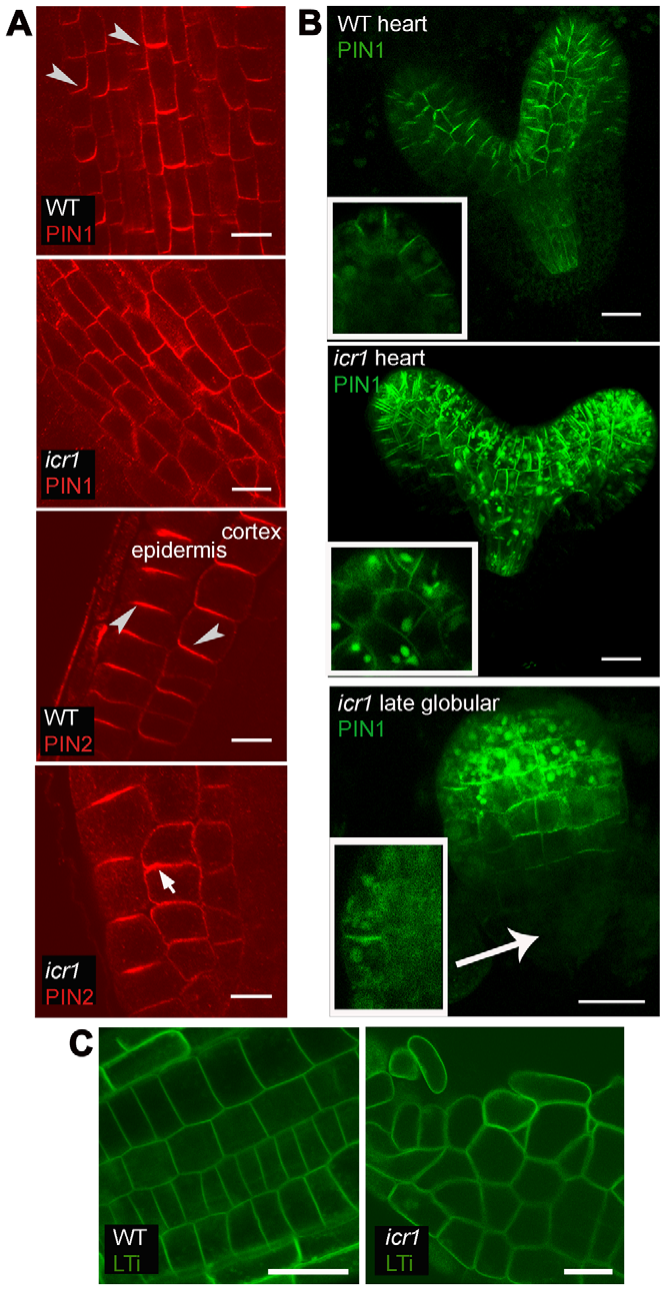
ICR1 is required for PIN polarity and membrane localization. (A) Indirect immunofluorescence of roots with α-PIN1/PIN2 antibodies. Arrowheads denote the direction of PIN polarity. Note the changes in polarity of PIN1 in provascular tissue and of PIN2 in the cortex (arrow). (B) Localization and expression pattern of *pPIN1*::GFP-PIN1 in embryos. Projection of multiple confocal sections shows that the PIN1 polarity in provascular tissue and protoderm in heart-stage *icr1* embryos is altered. Arrow indicates no expression at the basal side of the *icr1* embryo with early patterning defects. Insets are single confocal scans throughout the region of future cotyledons. Note the reduced PIN1 polarity and large intracellular aggregations in heart stage *icr1* embryo and almost complete loss of PIN1 membrane localization in embryo with early patterning defects. (C) Localization of the plasma membrane marker LTi-GFP is similar in roots of WT and *icr1* seedlings. Bars correspond to 20 µm. For additional information and high resolution images, see Figures S8, S9, S10, S11.

To examine a possible mechanism by which ICR1 mediates auxin distribution, we examined the localization and expression of PIN1 and PIN2 auxin transporters in WT and *icr1* roots and embryos (Figure 5 and Figures S8, S9, S10, S11, S12). Immunolocalization analysis showed a defect in polar localization of PIN proteins, in more pronounced cases resulting in basal-to-apical shift of PIN1 in the stele and of PIN2 in the cortex of *icr1* roots (Figure 5A, see arrowheads, and Figure S8). Consistently, in approximately 90% of the cells, reduced association of PIN1-GFP with the plasma membrane and basal to apical shift of PIN2-GFP of the cells in the cortex were observed (Figures S9 and S10A). In the epidermis, PIN2 is primarily localized at the apical side of the cells and is resistant to BFA at this location [21],[31]. PIN2 remained associated with the apical side of the *icr1* root epidermis cells (Figures 5A and S10A), suggesting that ICR1 primarily interacts with a BFA sensitive PIN delivery pathway. In globular *icr1* embryos showing early developmental defects, GFP-PIN1 was not expressed at the basal pole, accumulated in large aggregates inside the cells, and was largely absent from the plasma membrane (Figure 5B, arrow, and Figure S11). In embryos with late developmental aberrations, obvious changes in PIN1-GFP localization started to appear at the early heart stage and were associated with intracellular PIN1-GFP aggregations and overall loss of polar membrane localization (Figure 5B and Figure S11). The differences between the immunolocalizations and the PIN1-GFP possibly reflect differences between embryo and root cells or higher stability GFP-PIN1. Alternatively, the changes in auxin distribution affect *pPIN1*-driven PIN1-GFP expression such that it accumulates at higher levels in certain cells. Importantly, localization of the plasma membrane marker LTi-GFP was not affected in *icr1* mutants (Figure 5C), suggesting that ICR1 does not regulate targeting of membrane proteins in general. The model in Figure S12 summarizes the effects of ICR1 on embryo patterning, PIN1 localization, and auxin distribution. The reduced polarity and membrane association of PIN1 in *icr1* embryos likely leads to defective polar auxin transport and thus to defects in formation of auxin maxima in embryonic root and cotyledons. The reduction in auxin levels is associated with inhibition of PIN1 expression in the provascular tissue. This leads, in turn, to failure of the auxin maximum formation causing a collapse of the root meristem.

### ICR1 Expression Pattern

**Figure 6:**
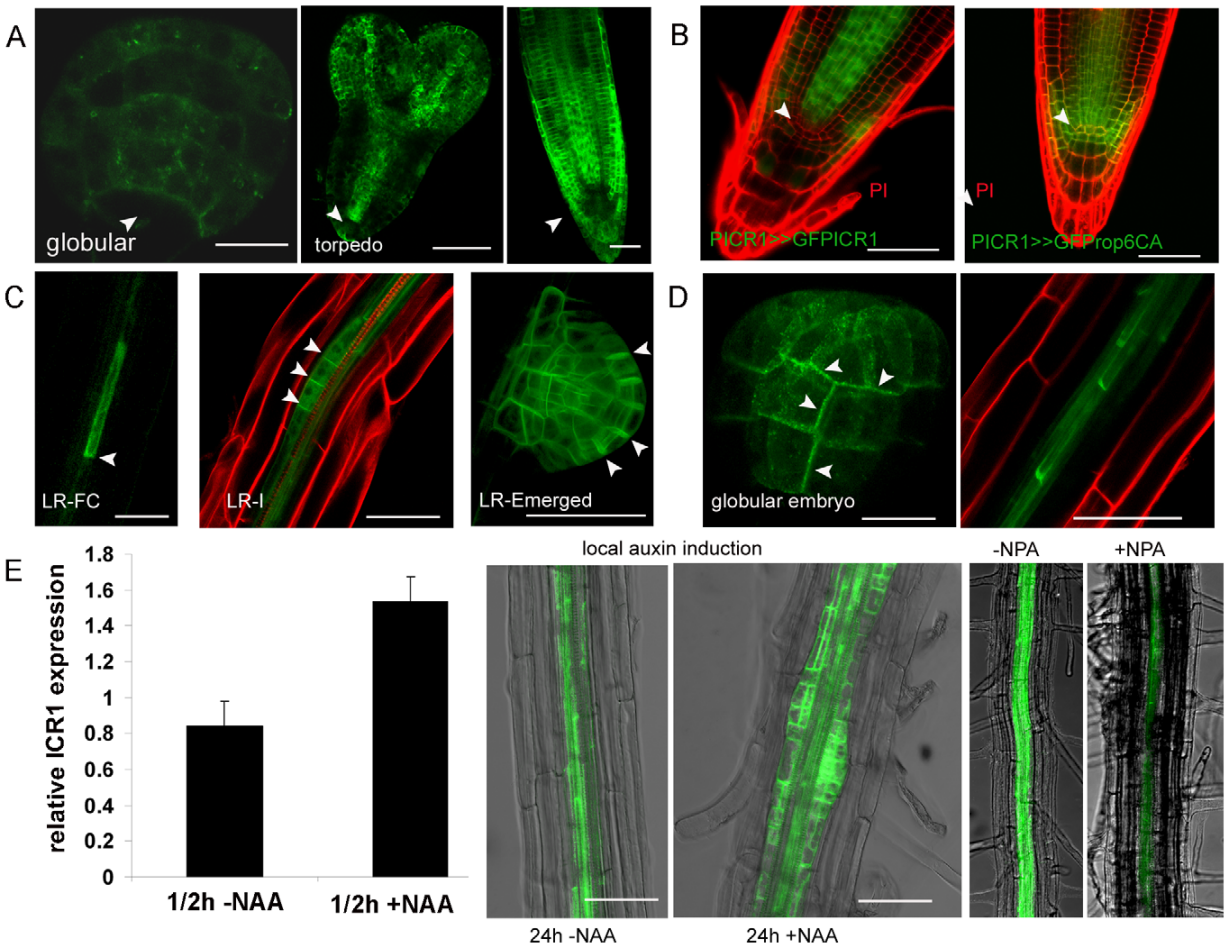
Expression of *ICR1* is induced by auxin but suppressed at the site of stable auxin maximum. (A) ICR1 expression in embryos and roots. In globular and torpedo stage embryos, GFP-ICR1 expression is absent from the hypophysis and QC, respectively. A projection stack of multiple confocal scans through mature roots shows GFP-ICR1 expression in the root cup and epidermis, but a partial projection stack through the inner layers only reveals that GFP-ICR1 is absent from the QC and neighboring cells (arrowheads). (B) Regulation of ICR1 expression involves the ICR gene and/or protein. *pICR1* driven GFP-rop6^CA^ but not GFP-ICR1 was expressed in the QC and stem cells (arrowhead). (C) ICR1 expression in lateral roots. GFP-ICR1 expression is detected in lateral root (LR) founder cells and throughout LR development. Note the polarized localization of the GFP-ICR1 (arrowheads). (D) Subcellular localization of GFP-ICR1. Plasma membrane and polarized GFP-ICR1 localization in globular embryos GFP-ICR1 is detected in basal and periclinal membranes (arrowheads). In roots, GFP-ICR1 becomes progressively polarized as cells in the stele mature. (E) *ICR1* expression is induced by auxin. Bar graph Q-PCR analysis showing induction of *ICR1* expression within 30 min of incubation with auxin (10 µM NAA). Strong induction of *pICR1*-driven GFP-ICR1 expression detected 24 h after local auxin induction with 1 mm^2^ solid-medium particles with or without 10 µM NAA that were put 5 mm above the root tip. *pICR1*-driven GFP-ICR1 expression was suppressed by treatment with the auxin transport inhibitor NPA. Bars correspond to 10 µm for globular embryos (A and D) and 50 µm in all the other images. For additional information and high resolution images, see Figures S13, S14, S15, S16.

An auxin response element (GTGCTC), which is located 254 base pairs (bp) upstream of the initiation AUG codon of *ICR1* (Figure S13), indicated that ICR1 could be part of an auxin-induced positive feedback loop that influences polarity of auxin transport. It further suggested that ICR1 might integrate nuclear auxin signaling [32] and ROP-regulated cell polarity. To examine these hypotheses, we studied the ICR1 expression pattern and subcellular localization using a genomic clone of *ICR1* fused to *GFP* (Figure 6 and Figure S13). In globular and torpedo stage embryos, GFP-ICR1 expression was observed throughout the embryo proper but interestingly not in the hypophysis and QC where a stable auxin maximum is formed (Figure 6A). Similarly in roots, ICR1 was absent from the QC and the stem cells, positions of stable auxin maxima (Figure 6A and 6B and Video S1). The absence of ICR1 expression at the position of the auxin maxima correlated with non-polar PIN4 localization in the hypophesis, QC, and columella initials [33].

Several experiments were carried out to examine whether ICR1 expression is influenced by auxin. Expression analysis by quantitative real-time RT-PCR (q-PCR) showed that ICR1 expression is quickly induced following 30 min of incubation in auxin (NAA) (Figure 6E). The expression of *pICR*≫*GFP-ICR1* and *pICR1*≫*GUS* reporters following growth of seedling on medium containing the polar auxin transport inhibitor NPA (1-N-naphthylphthalamic acid) and followed by addition of auxin (NAA) to the growth medium were studied. Confirming the prediction, ICR1 expression was lower when seedlings were grown on NPA and was induced as early as 4–6 h following treatment with NAA (Figure 6E and Figure S14). As shown above, lateral root development is compromised in *icr1* (Figure 4E). The initiation of lateral roots is induced by auxin and occurs at places of transient local auxin maxima [15],[28]. Consistently, GFP-ICR1 expression was observed at positions of lateral root initiation (Figure 6C), further indicating that ICR1 expression is induced by auxin.

The absence of ICR1 in the QC and stem cells, where the stable auxin maximum is formed, was in apparent discrepancy to the induction of its expression by auxin and suggested that it might be suppressed by a different mechanism. To examine whether a stable auxin maximum could suppress *ICR1* expression directly, auxin was applied locally to *pICR1*≫*GFP-ICR1* roots, using small, 1 mm^2^, agar particles that contained 10 µM NAA. Strong local GFP-ICR1 expression was observed in the auxin treated roots, while no increase was observed in control roots that were treated with agar particles without auxin (Figure 6E). These results reconfirmed that *ICR1* expression is induced by auxin and further suggested that stable auxin maxima, likely, do not suppress ICR1 expression directly. The site of the stable auxin maximum at the root tip was shown to express a specific group of genes [18],[27],[34]. Thus, possibly, the suppression of ICR1 expression is associated with specific cellular processes taking place at and around the site of the stable auxin maxima.

To obtain further insight into regulation of ICR1 expression, we examined whether the suppression of its expression may be associated with the ICR1 gene or protein. To this end, a GFP-rop6^CA^ reporter (Poraty and Yalovsky, unpublished data) was expressed under regulation of the *ICR1* promoter. To reduce the possibility of differences in expression due to position effects, expression was carried out using the LhG4/pOp transcription/transactivation system [35],[36], using the same *pICR1* promoter activator lines to express GFP-rop6^CA^, GUS, or GFP-ICR1 (Figure S13). In contrast to GFP-ICR1, the GFP-rop6^CA^ was observed in the QC and stem cells (Figure 6B), indicating that indeed the suppression of ICR1 expression in the root meristem could be regulated by a mechanism associated with the ICR1 gene or protein. To summarize these data, while auxin induced transcription of *ICR1* is part of a positive feedback loop that facilitates auxin transport, the suppression of ICR1 expression in the root meristem is associated with a cell-specific mechanism/s, presumably leading to inhibition of directional auxin transport, and contributes to the formation of a stable auxin maximum.

### Subcellular Localization of ICR1

**Figure 7:**
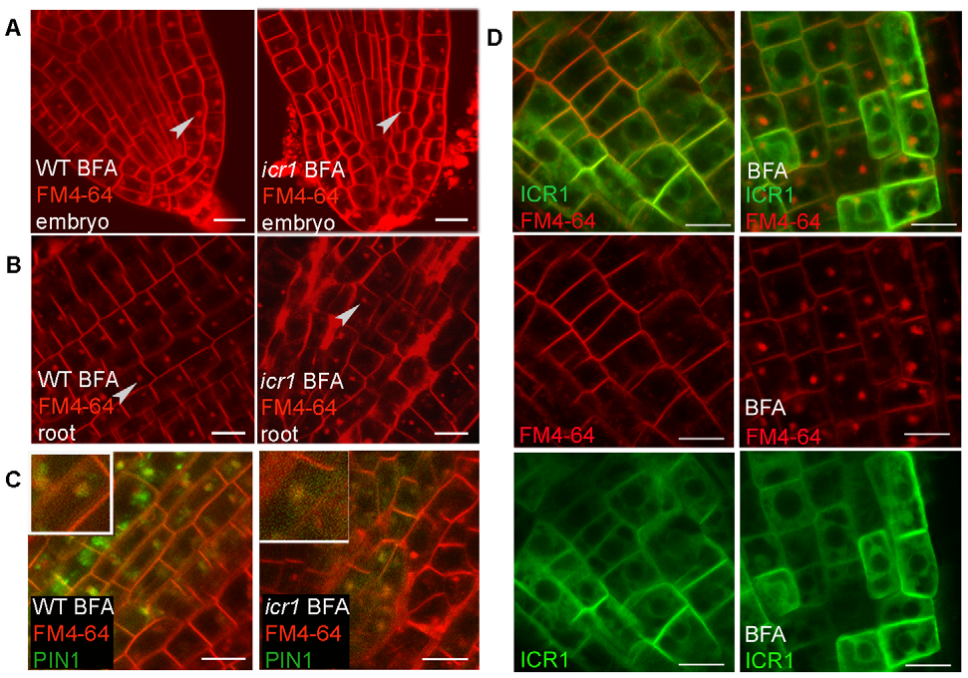
BFA-sensitive endocytic recycling is not affected in *icr1* and GFP-ICR1 localization is insensitive to BFA. (A, B) BFA treatments of embryos and roots stained with FM4-64, respectively. Arrowheads mark BFA compartments. Note that in *icr1* BFA compartments are formed in all root tissues examined. (C) *pPIN1*::GFP-PIN1 co-localizes with FM4-64 in BFA compartments of WT and *icr1* (arrowheads) root cells. (D) *pICR1* driven GFP-ICR1 does not accumulate in FM4-64-labelled BFA bodies and its membrane localization is not affected by BFA treatments. Bars correspond to 10 µm (A) and 20 µm (B to D). For additional information, see Figure S10B.

Next, we examined whether the subcellular localization of GFP-ICR1 reflects ICR1 function in polar localization of PIN proteins. GFP-ICR1 complemented root growth in *icr1* mutant plants (Figure S15). Furthermore, in pollen tubes, overexpression of either GFP-ICR1 or non-fused recombinant ICR1 had the same phenotypic effects [13]. It thus appears that localization of the GFP-ICR1 fusion protein reflected that of the native ICR1 protein. Importantly, GFP-ICR1 localization was not sensitive to BFA (Figure 7D), consistent with earlier studies showing that recruitment of ICR1 to the plasma membrane is not ARFGEF- but ROP-dependent [12],[13]. GFP-ICR1 was localized both at the plasma membrane and intracellularly. Plasma membrane localization was confirmed by plasmolysis, which induces detachment of membrane from the cell wall (Figure S16). In lateral root founder cells and embryos, GFP-ICR1 had polarized localization (Figure 6C and 6D, arrowheads). In the primary root, the localization of GFP-ICR1 became progressively polarized away from the auxin maximum (Figure 6D, arrowheads). The non-polarized ICR localization around the auxin maximum could contribute to the reduction of auxin transport leading to the formation of stable auxin maximum.

### ICR1 and Constitutive Endocytic Recycling

The reduced polarity and association with the plasma membrane of PIN1 and PIN2 in the *icr1* plants suggested that ICR1 is required for recruitment of the PIN proteins to the plasma membrane rather than their Rab5-regulated endocytic recycling [37]. Indeed, internalization of endocytosis tracer FM4-64 into BFA bodies occurred normally in *icr1* mutant roots and embryos (Figure 7A and 7B). Furthermore, similar to WT roots, PIN1-GFP and PIN2-GFP were internalized into the BFA bodies in *icr1* roots (Figures 7C and S10B). These data confirmed that endocytosis of FM4-64-labeled plasma membrane derived vesicles in general and of PIN1 and PIN2 in particular were not affected in *icr1* mutant plants.

**Figure 8:**
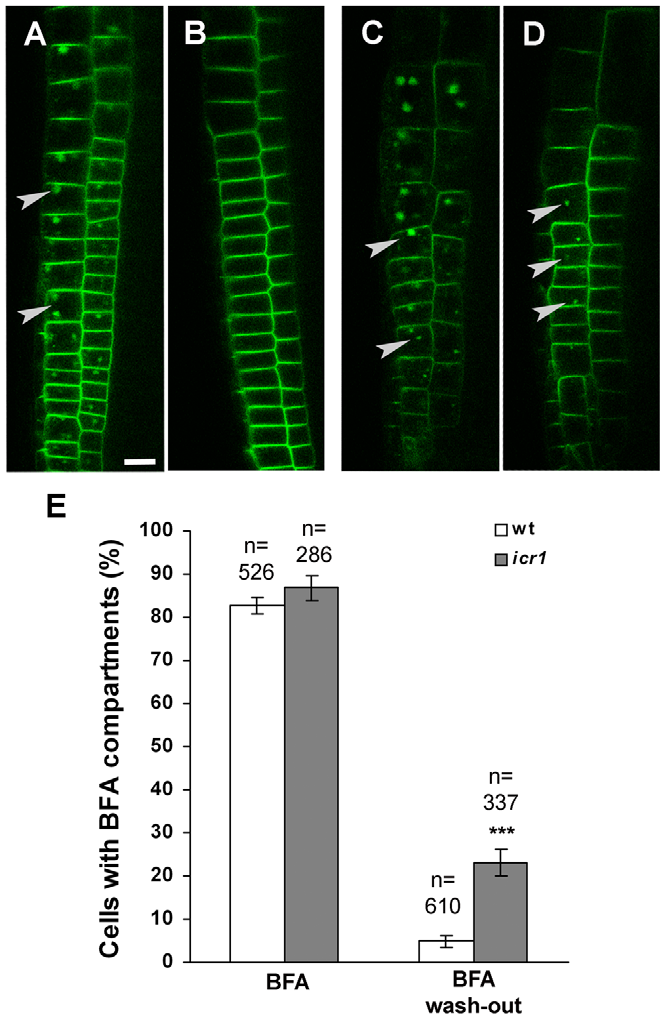
Stability of PIN2-GFP labeled BFA bodies in WT and *icr1* roots. PIN2-GFP labeled BFA compartments in *PIN2-GFP* (A) and *PIN2-GFP icr1* (C) epidermal and cortical layers treated with 50 µM BFA for 1 h *PIN2-GFP* (B) and *PIN2-GFP icr1* (D) after 2 h BFA washout. (E) Percentage of epidermal cells with BFA bodies before and after BFA washout in *PIN2-GFP* and *PIN2-GFP icr1*. Error bars indicate SE; *** *p*≤0.001; Student's *t* test. Arrowheads mark BFA bodies. The scale bar corresponds to 10 µm for all images.

The effects of BFA on endocytic recycling are reversible. When BFA is washed from the medium, the BFA bodies disappear and PIN proteins regain their localization in the plasma membrane [21],[22]. BFA washout experiments were carried out to examine whether PIN dynamics is compromised in *icr1*. Following incubation of *GFP-PIN2* and *GFP-PIN2 icr1* plants in BFA, the GFP-PIN2-containing BFA bodies appeared in 80% to 90% of the cells and no significant differences were found between WT and *icr1* (Figure 8A, 8C, and 8E). However, following a 2-h incubation in medium without BFA, 20%–27% of the cells in the *icr1* roots still contained BFA bodies compared to only 2%–5% of the cells in WT plants (Figure 8B, 8D, and 8E). The differences between WT and *icr1* roots in the percentage of BFA bodies containing cells were statistically significant (*p*≤0.001; Student's *t* test). These results reconfirmed that ICR1 is likely not involved in the Rab5-mediated endocytosis accumulation of PIN proteins following BFA treatments and indicated that ICR1 affects the recycling of PIN proteins back to the plasma membrane.

### Function of ICR1 in Exocytosis

**Figure 9:**
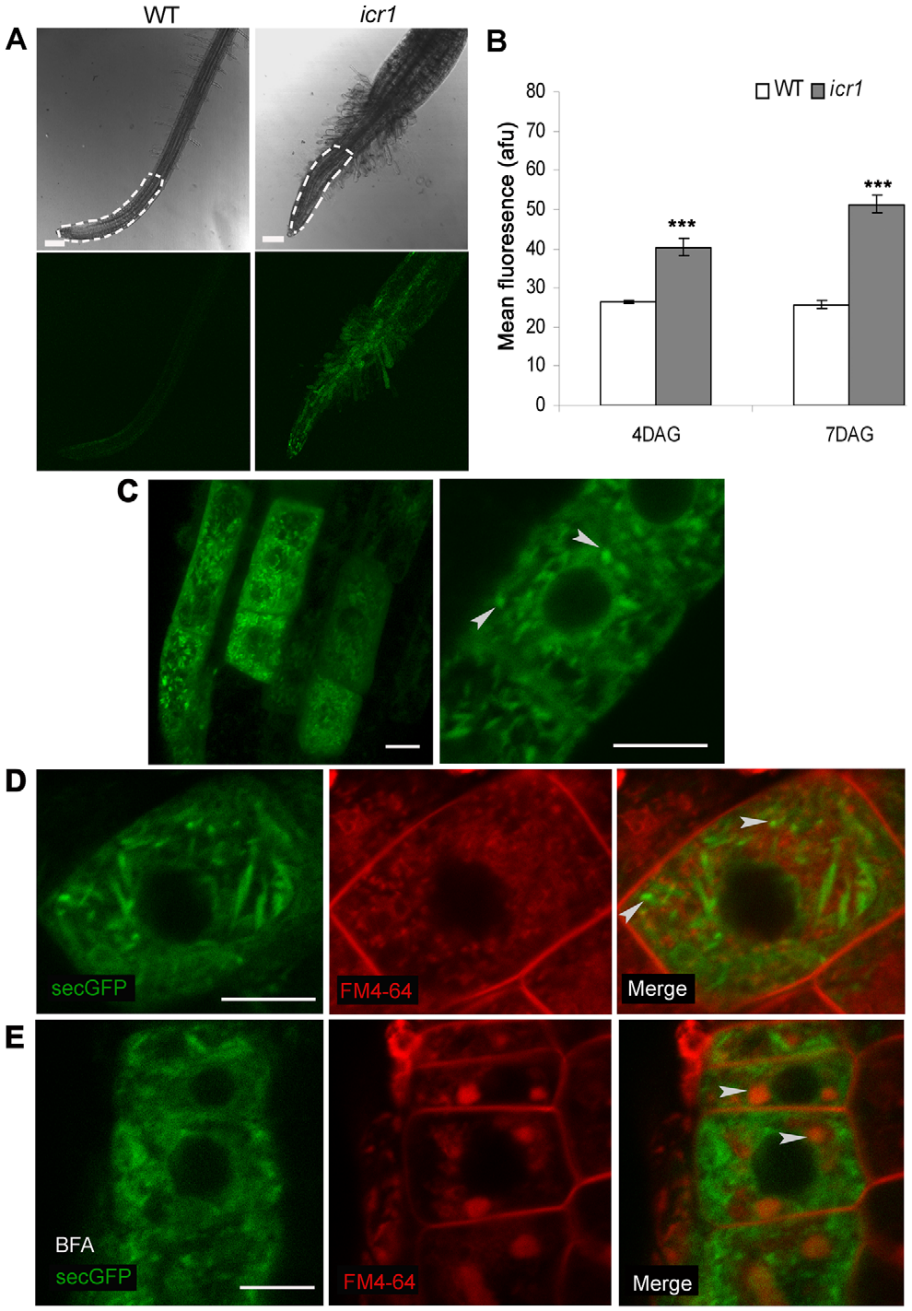
Localization of secGFP in *icr1* roots. (A) Representative secGFP fluorescence images (two bottom panels) of WT and *icr1* roots at 4 DAG imaged under identical conditions. White dotted lines on the DIC images (two upper panels) mark an area of 500 µm in length from the root tip that was used for quantification of the mean fluorescence shown in (B). (B) Mean fluorescence in WT and *icr1* roots at 4 and 7 DAG. The mean fluorescence in *icr1* roots was ∼1.5-fold stronger at 4 DAG and ∼2-fold stronger at 7 DAG. afu, arbitrary fluorescence units. Error bars correspond to SE; *n*≥20; *** fluorescence intensity was significantly different between WT and *icr1* roots (*p*≤0.001; Student's *t* test). (C) Localization of secGFP in *icr1* roots at 4 DAG. Note the accumulation of secGFP in punctuate structures (arrowheads). (D) secGFP (green) punctuate structures in *icr1* were not co-localized with early/recycling endosomes marked with FM4-64 (red) (arrowheads). (E) secGFP (green) is not internalized into the BFA compartments marked by FM4-64 (red, arrowheads). Bars correspond to 100 µm in (A) and 10 µm in (C to E).

Previously, we showed that ICR1 interacts with AtSEC3A exocyst complex subunit and that ROPs can recruit ICR1-SEC3 complexes to the plasma membrane [12]. This suggested that ICR1 could be involved in regulation of polarized exocytosis. To test this hypothesis, comparison of the distribution of a protein secretion marker, secGFP, in WT and *icr1* mutant plants was carried out. SecGFP is a secreted form of GFP that is fused to the chitinase signal peptide at its N-terminus and to HDEL, ER-retention signal, at its C-terminus and has been used in analysis of protein trafficking [38],[39]. Secretion of secGFP to the apoplast results in weak signal due to the relatively acidic pH of this compartment. Perturbation of secretion results in fluorescence of intracellular accumulating protein. Thus, the effect of a given mutant or treatment on secretion can be evaluated by monitoring the differences in fluorescence. Transgenic plants expressing secGFP [38],[39] were crossed into the *icr1* background. Qualitative and quantitative fluorescence imaging analyses showed that the GFP fluorescence in *icr1* roots was significantly stronger (*p*≤0.001; Student's *t* test) than in WT roots (Figure 9A and B). Fluorescence of secreted GFP can be recovered when seedlings are grown at a pH value higher than 8.1 [39]. To validate that the differences in GFP fluorescence between WT and *icr1* roots were associated with protein secretion rather than an unrelated mechanism, seedlings were transferred from low (pH 5.5) to high (pH 8.5) pH medium. Quantitative analysis showed significant (*p*≤0.01; Student's *t* test) increase in GFP fluorescence of WT roots, while no increase was observed in *icr1* roots (Figure S17). These data confirmed that the secGFP secretion was compromised in *icr1*. High magnification fluorescent confocal images showed that in *icr1* plants secGFP accumulated in punctuate structures (Figure 9C). The internalized secGFP was not co-localized with FM4-64-labeled intracellular vesicles (Figure 9D) and did not accumulate in BFA compartment (Figure 9E).

## Discussion

Our results show that ICR1 regulates directionality of polar auxin transport and is thus required for the formation of a stable auxin maximum and tip localized auxin gradient during embryogenesis, organogenesis, and meristem activity. Earlier work on auxin related patterning enforced the notion that patterns do not reflect a rigid program but rather the inherent developmental potential of each cell [30]. Results presented in this work imply that ICR1 is part of an auxin regulated positive feedback loop, integrating auxin-dependent transcriptional regulation with Rho family GTPases-mediated modulation of cell polarity. Thus, ICR1 forms an auxin-modulated link between cell polarity and auxin transport-dependent tissue patterning.

Dynamic PIN polarity is achieved by (1) integration of a previously described constitutive endocytic recycling that involves clathrin-dependent endocytosis [14],[23] and BFA-sensitive ARF GEFs-mediated recycling [21],[22] and (2) a ROP-ICR1-regulated, BFA-insensitive exocytosis (this work). ROP-ICR1 complexes were detected in the plasma membrane [12],[13]. Furthermore, following plasmolysis a substantial fraction of *pICR1* driven GFP-ICR1 remained associated with the plasma membrane (Figure S16). Thus, it is likely that ICR1 functions in conjunction with ROPs to recruit PIN proteins to specific sites in the membrane. Future analysis of PINs dynamics, using techniques such as Fluorescence Recovery After Photobleaching (FRAP) [37], would be required to elucidate the interactions between ICR1-associated exocytosis and the BFA-sensitive PIN endocytic recycling.

The exocytosis defects in *icr1* are consistent with a previous finding that ICR1 can interact with both ROPs and AtSEC3A exocyst subunit at the plasma membrane [12]. Plants have an evolutionarily conserved exocyst complex [40] that based on mutational analysis was implicated in regulation of polarized secretion, cell morphogenesis, and patterning [40]–[43]. The reduced association of PINs with the membrane, their accumulation inside the cells in the *icr1* mutant background (Figure 5 and Figures S8, S9, S10, S11), and the slower recruitment of PIN2 to the plasma membrane following BFA washout (Figure 8) indicate that ICR1 and possibly exocyst-dependent exocytosis acting at or close to the plasma membrane is required for proper PIN localization. Thus, in the absence of ICR1, due to endocytic recycling, the PIN proteins are degraded or accumulate in the cells. Basal to apical shift of PIN1 and PIN2 have been associated with function of PINOID (PID) protein kinase as well as a BFA-insensitive pathway [14],[31]. The basal to apical shift of PIN1 and PIN2 that were observed in *icr1* suggests that ICR1 has either reduced or no effect on this BFA insensitive pathway.

ROPs are recruited to the plasma membrane by virtue of the posttranslational lipid modifications prenylation or *S*-acylation that take place on conserved C-terminal cysteine residues [1],[7],[8],[9],[44]. In addition, at least some ROPs also undergo activation-dependent transient *S*-acylation and consequent partitioning into detergent resistant membranes that could be lipid rafts [9]. Association with the inner leaflet of the plasma membrane also requires a polybasic domain proximal to the lipid modified cysteines [8]. It has been shown that the polybasic domain in Rho proteins associates with phosphatidylinositol 4,5-bisphosphate (PtdIns-P2) and phosphatidylinositol 3,4,5-trisphosphate (PtdIns-P3) [45]. Consistently, in pollen tubes tip, ROP/RAC proteins were shown to physically associate with a phosphatidylinositol monophosphate kinase (PtdIns P-K) activity [46], and PtdIns-P2, the product of PtdIns P-K, had similar distribution [46],[47]. When expressed in pollen tubes, ICR1/RIP1 and ROP1 stabilized the membrane localization of each other at the growing tip, suggesting that ICR1 may interact with other components in the membrane in addition to ROPs [13]. It appears therefore that determination of ROP-ICR1 subcellular localization involves multiple mechanisms, including different protein lipid modifications, partitioning into discrete membrane microdomains, and association with phosphatidylphosphoinositides and possibly with yet unidentified components.

At the root tip, localization of GFP-ICR1 became progressively associated with the plasma membrane and polarized in cells that were more distantly located away from the stable auxin maximum (Figure 6D). This suggests that membrane localization and polarization of ICR1 are locally regulated, possibly by a local auxin gradient associated mechanism.

Auxin modulates its polar efflux from cells by inhibiting PIN endocytosis [48] and possibly by regulating the expression of ICR1 (this work). Thus, ICR1 may be part of an auxin modulated positive feedback loop that facilitates auxin efflux. ICR1 expression in the root (Figure 6) coincides with pattern of auxin flux and with non-polar localization of PIN4 in the sites of the auxin maxima in the hypophesis, QC, and columella initials [14],[33]. In most regions of the primary root, the expression of ICR1 is limited to the stele (Figure 6). The expression pattern changes in two regions: (1) lateral root founder cells and initials and (2) around the root tip where expression is detected in epidermis, cortex, endodermis, and the root cap. The stable auxin maximum and local gradient at the root tip are associated with expression of specific genes. The expression level of some of these genes has been shown to correlate with auxin levels [27],[34],[49]. It is likely that the repression of ICR1 expression in the QC and the meristematic stem cells is associated with one or some of these auxin maximum-specific genes. The quick induction of *ICR1* expression by auxin and the strong expression of *pICR1*-driven GFP-ICR1 by local application of auxin further suggest that suppression of ICR1 expression at the site of auxin maxima is indirectly regulated by auxin. Interestingly, mutants in the *HALTEDROOT* (*HLR*) gene, which encodes an RPT2a 26S proteasome subunit, share a similar phenotype with *icr1*. Like *icr1*, the root meristem of *hlr* collapses, the QC is lost, and expression pattern of several markers including *SCR* and *SHR* is altered [50]. In contrast to *icr1*, however, additional tiers of columella cells, compared to WT, were observed in 6-d-old *hlr* seedling, while in *icr1* plants, of similar age, the specification of the columella is significantly reduced (Figure 2). No differences in the *icr1* expression pattern were observed when *pICR1*≫*GFP-ICR1* plants were treated with a proteasome inhibitor (unpublished data). Thus, it is currently unclear whether the proteasome is involved in regulation of ICR1 expression.

Based on computational modeling, it has been proposed that the PIN-mediated auxin fluxes in the root are sufficient for maintaining a stable auxin maximum [51]. Results of this work (Figure 6) implicate the repression of ICR1 expression and possibly also regulation of its subcellular localization in the formation of the stable auxin maximum. The stable auxin maximum may facilitate a positive feedback loop that maintains the repression of ICR1 expression and its distribution in the immediate proximal and subtending cells.

Constitutive active ROP-induced cell deformation has been associated with reorganization of actin and MT cytoskeleton, as well as compromised vesicle uptake at the plasma membrane [1],[2]. Ectopic expression of ICR1 induced deformation of leaf epidermis pavement cells and root hairs [12] as well as pollen tubes [13], resembling the effect of activated ROP mutants. Thus, ICR1 may act as a scaffold that facilitates compartmentalization of ROPs and other proteins such as the exocyst complex at specific membrane domains [12]. This also suggests that ICR1 could be involved in regulation of various processes.

ROPs orchestrate cell polarization by regulating cytoskeleton organization through proteins that include RIC1 (ROP Interacting CRIB containing 1), RIC3 and RIC4 [52],[53], ADF/Cofilin [54], and the WAVE and Arp2/3 complexes [55],[56]. As shown in this work and based on previous findings [12], the ROP-ICR1-associated cell polarization machinery is required for plasma membrane recruitment and polar localization of PIN proteins, consequently directing auxin transport.

## Materials and Methods

### Molecular Cloning

#### *PICR1::LhG4*

The *ICR1* promoter was cloned by amplifying a 2,050 bp fragment upstream of the initiation ATG codon from genomic DNA with the oligonucleotide primers SYP1502 (CCGGTACCTTTGATTTCGTGTTGAGG) and SYP1503 (CGTGTCGACCCTCCTACAGAAGGTTGG) and cloned into pGEM (Promega). The resulting plasmid (pSY1500) was digested with Kpn1 and Sal1, and the resulting fragment was subcloned into pLhG4-Bj36 upstream of the synthetic transcriptional factor gene *LhG4* [35],[36] to yield pSY1501. pSY1501 was digested with Not1, and the *pICR1-LhG4* fragment was subcloned into the plant binary vector pART27 to yield pSY1502.

#### *pOp::GFPICR1*

A 2,339 bp genomic fragment of *ICR1*, including the entire 5′-UTR of the gene, exons, introns, and the 3′-UTR, was cloned into the plant binary vector pMLBART as follows: the *ICR1* 5′-UTR was amplified from genomic DNA by PCR using oligonucleotide primers SYP321 (CAGCCATGGGACGTCGACATTTGATCAGC) and SYP322 (ATTATATCCTCAACACGAAATCAAACCATGGCTG), digested with NcoI and subcloned upstream of *GFP* in pGFP-MRC [57] to yield pSY350. The genomic *ICR1* gene starting from the initiating ATG codon was amplified by PCR from genomic DNA using primers SYP323 (CTAGAGCTCATGCCAAGACCAAGGTTACG) and SYP324 (GCAGGTACCGTTTAACGGGTTTCTCCATTTACGA), digested with SacI and KpnI and subcloned into pSY350 downstream of *GFP* to yield pSY351. pSY351 was in turn digested with SalI and KpnI, and the resulting fragment containing *pICR1-5′UTR′-GFP-ICR1_genomic_-ICR1-3′UTR* was subcloned into pOPTATABJ36 to yield pSY352. pSY352 was in turn digested with NotI, and the resulting fragment was subcloned into the plant binary vector pMLBART to yield pSY353.

#### *pOp::His_6_-GFP-rop6^CA^*

The pSY812 plasmid containing *His_6_-GFP-rop6^CA^* was prepared as previously described [9]. pSY812 was digested with XhoI. The resulting fragment containing *His_6_-GFP-rop6^CA^* was subcloned into p10Op-Bj36 [36] to obtain pSY819. In turn, pSY819 was digested with NotI, and the resulting fragment containing 10*Op-His_6_-GFP-rop6^CA^-OCS* terminator was subcloned into the plant binary vector pMLBART [35],[36] to obtain pSY818. Expression in the transcription-transactivation system [35],[36] was achieved by crossing the *promoter::LhG4* activator lines with the respective *pOp::reporter* lines.

### q-PCR

Total RNA was isolated with “SV Total RNA isolation” according to the manufacturer's instructions (Promega, Madison). cDNA first strand synthesis was carried out using Super ScriptII reverse transcriptase (Invitrogen Carlsbad, USA). Quantification with the oligonucleotide primer set SY1582: TCAAAATGCCAAGACCAAGA and SY1583: TTGGAATGATTGGAATCAGAAG was carried out by q-PCR using an ABI Prism 7700 StepOnePlus™ Instrument (Applied Biosystems, Weiterstadt, Germany). Study samples were run in triplicate on 8-well optical PCR strips (Applied Biosystems) in a final volume of 10 µl. Primers were designed using Roche Universal Probe Library (https://www.roche-applied-science.com/sis/rtpcr/upl/index.jsp). The PCR cycles were run as follows: 10 min initial denaturation at 95°C, followed by 40 subsequent cycles of 15 s denaturation at 95°C, and 1 min annealing and elongation at 60°C. The specificity of the unique amplification product was determined by a melting curve analysis according to the manufacturer's instructions (Applied Biosystems, Weiterstadt, Germany). Relative quantities of RNA were calculated by the ddCt method (Applied Biosystems Incorporated (2001), User Bulletin #2: ABI PRISM 7700 Sequence Detection System, http://www.appliedbiosystems.com). cDNA dilution series were prepared to calculate amplification efficiency coefficient. The relative levels of RNA were calculated according to the amplification efficiency coefficient and normalized against an *UBQ21* gene standard [58], whose level was taken as 1. The stability of the standard in each experiment was verified with the geNorm analysis tool (http://medgen.ugent.be/jvdesomp/genorm/) and was calculated as *M*≤0.7. The analysis was repeated with three independent biological replicates.

### Plant Growth Conditions

Seeds of WT *Columbia-0* (*Col-0*) and mutant Arabidopsis plants were sown on soil (Universal potting soil, Tuff Moram Golan, Israel) and left for 2 d at 4°C. Then, plants were transferred to a growth chamber and were grown under long-day conditions (16 h light/8 h dark cycles) at 22°C. The light intensity was 100 µE m^−2^ s^−1^. For seedling analysis, seeds were surface sterilized and sown on plates containing 0.5× Murashige & Skoog (0.5× MS) salt mixture (Duchefa) titrated to pH 5.5 with MES and KOH, 1% sucrose, and 0.8% plant agar (Duchefa) and left for 2 d at 4°C. Then plates were transferred to the growth chamber and grown under long-day conditions (16 h light/8 h dark cycles) at 22°C for an indicated period. The light intensity was 100 µE·m^−2^·s^−1^. For auxin induction experiments, seedlings were germinated on plates (as above) for 5 d, then transferred to liquid medium (0.5× MS), and grown for 2 additional days before application of either NPA (10 µM) or NAA (10 µM).

### Drug and Dye Treatments and Plasmolysis

#### BFA (Fluka) treatments

Two to 3-d-old seedlings were submerged in 25, 50, or 90 µM BFA (prepared from a 100 mM stock solution in DMSO) and 4 µM FM4-64 (prepared from a 1 mM stock solution in DMSO) diluted in 0.5× MS liquid medium for 1–2 h at room temperature. For control treatments, seedlings were submerged in 0.09% DMSO solution containing 4 µM FM4-64. For BFA treatments of embryos, heart and torpedo stage embryos were dissected from ovules under stereo-microscope and immediately transferred into 0.5× MS liquid media supplemented with 1% sucrose. Dissected embryos were treated with 50 µM BFA and 5 µM FM4-64 on glass slides for 1–2 h in dark and observed with a 63× water-dipping (cover slide-free) objective using the Confocal Laser Scanning Microscope (CLSM).

#### Propidiumiodide (PI) staining

Seedlings were in 50 µg/ml PI in water solution submerged on microscope slides.

Plasmolysis was carried out by incubating detached leaves in 0.8 M NaCl for 5 min [7].

### GUS Staining

β-Glucoronidase (GUS) staining was carried out as previously described [59], except that seedling were submerged in the staining solution for 2 h and then clarified in either chloralhydrate/glycerol/water (8∶1∶2) or 70% ethanol.

### Clearing of Arabidopsis Embryos

Clearing of Arabidopsis embryos was performed as previously described [60]. In short, growing siliques were harvested from soil-grown plants and dissected under a stereo-microscope. Ovules from individual siliques were collected and fixed for 1–4 h in ethanol/acetic acid (6∶1) at room temperature. Then, ovules were washed three times for 5 min in 100% ethanol and one time in 70% ethanol. In turn, the ovules were incubated in a clearing solution (chloralhydrate/glycerol/water 8∶1∶2 v/v) for 24 h, mounted on slide with 30% glycerol, and observed by Nomarsky Differential Interference Contrast (DIC) optics.

### Starch Staining

Seedlings of indicated age were transferred into Lugol (IKI—0.2% w/v iodine and 2% potassium iodine) and incubated for 3 min. The Lugol-stained seedlings were then briefly washed with water and mounted on the slide with a clearing solution (chloral hydrate in 30% glycerol).

### Local Auxin Induction

Small solid medium (0.5× MS, 1% sucrose, neutral red, 0.8% plant agar) particles, approximately 1 mm^2^ in diameter, with or without 10 µM NAA were applied onto roots of vertically grown 7-d-old *pICR1*≫*GFP-ICR1_genomic_* seedlings 5 mm above the root-tip. GFP-ICR1 expression was observed 24 h after the treatment.

### Light and Confocal Laser Scanning Microscopy

Nomarsky/DIC imaging was performed with an Axioplan-2 Imaging microscope (Carl Zeiss, Jena, Germany) equipped with an Axio-Cam, cooled charge-coupled device (CCD) camera by using either 40× or 100× oil immersion objectives with numerical aperture (NA) values of 1.3 or a 63× water immersion objective with NA value of 1.2. Low magnification imaging was carried out with Olympus MVX10 fluorescence stereomicroscope. CLSM imaging was performed with Leica TCS-SL CLSM with 20× multi-immersion, 63× water, and 63× water dipping (cover slide-free) objectives with NAs of 0.7, 1.2, and 0.9, respectively.

#### Visualization of fluorescent markers

GFP was visualized by excitation with an argon laser at 488 nm. Emission was detected with a spectral detector set between 505 and 530 nm. YFP was visualized by excitation with an Argon laser at 514 nm and spectral detector set between 525 and 560 nm for detection of emission. FM4-64 was visualized by excitation with an argon laser at 514 nm. Emission was detected with a spectral detector set between 530 and 560 nm. PI was visualized by excitation at 514 nm. The emission was detected with the spectral detector set between 600 and 650 nm.

### BFA Washout Assays

Four-day-old *GFP-PIN2* and *GFP-PIN2 icr1* seedlings were treated with 50 µM BFA for 1 h and then washed with 0.5× MS medium for 2 h. For calculation of epidermal cells with BFA bodies, at least 15 roots were used for each line and treatment. The experiment was repeated three times. Statistical analysis was performed with Student's *t* test.

### Secretion Assays

Analysis of secretion was performed as previously described [38],[39]. Transgenic Arabidopsis homozygous line expressing *35S::secGFP* in the *Col-0* background was crossed to *icr1*. T2 progeny homozygous for both *icr1* and secGFP were selected. Seedlings were grown on 0.5× MS plates (pH 5.5) supplemented with 1% sucrose for the indicated time. Images of WT and *icr1* root tips were taken with Leica LCS LSCM under identical conditions using a 10× objective, fully opened pinhole (600 µm) and emission/excitations of GFP as described above. The mean signal intensity was measured on an area spanning up to 500 µm from the root tip, using Image-J. DIC images of the same root were used for the adjustment of the measured area. At least 20 seedlings of each line were used in each experimental repeat. Statistical analysis was performed with Student's *t* test.

#### Effect of external pH on secGFP fluorescence

For comparison of secGFP fluorescence at external pH values of 5.5 and 8.5, seedlings were grown for 5 d on 0.5× MS plates pH 5.5, supplemented with 1% sucrose, and in turn transferred for 3 h to 0.5× MS liquid medium titrated to pH 5.5 or 8.5 with MES or KOH and supplemented with 1% sucrose.

## Supporting Information

Figure S1 **Abnormal development of early** ***icr1*** **embryos.** (A–F) *Col-0*, (G–L) 90% of *icr1* progeny with normal development at early embryo development, (M–P) 10% of *icr1* progeny with early basal defects. (A and G) 1-cell stage, (B and H) 4-cell stage, (C, I, and M) 8-cell stage, (D, J, and N) 16-cell stage, (E, K, and O) early globular stage, and (F, L, and P) late globular. h, hypophysis; lc, lens-shaped cell; llc, large lower cell. Arrowheads in (M and N) mark abnormal division in hypophysis and suspensor; vertical brackets in (O and P) mark unshaped basal region. Bars correspond to 10 µm for all images.
(1.88 MB TIF)

Figure S2 **Abnormal development of late** ***icr1*** **embryos.** (A–D) Triangular stage, (A) *Col-0*, and (B–D) *icr1*. Arrowheads in (B) indicate abnormal divisions in suspensor and columella initials; arrowhead in (C and D) marks abnormal division in protoderm. (D) Enlargement of the arrow-highlighted section in (C). (E–K) Early/mid-heart stage, (E and J) *Col-0*, (F–I and K) *icr1*. Arrowheads in (G–I) mark abnormal divisions in protoderm. Arrowhead in (J) indicates a normal periclinal division in a WT columella. Arrowheads in (K) point to abnormal division planes in columella initials of *icr1* embryos. (L–S) A bent cotyledon stage, (L, N, P, and R) *Col-0*, and (M, O, Q, and S) *icr1*. (N and O) Enlargement of the root meristem (RM). The arrowhead in (O) marks abnormal division in columella. Vertical bars in (L and M) indicate enlarged region shown in (P and Q). pd, protoderm; pc, procambium; col, columella initials; QC, quiescent center; v, vascular tissue; ep, epidermis; c, cortex; en, endodermis. Bars correspond to 20 µm (A–M), 10 µm (N–Q), and 50 µm (R and S).
(3.72 MB TIF)

Figure S3 ***icr1*** **siliques display greater developmental variability.** Stacked-bar charts show the developmental stages of embryos collected from 12 representative siliques. (A) *Col-0* and (B) *icr1* siliques of various ages. Each color marks a single silique. The high mixing of colors in *icr1* mutant indicates that the synchronization of embryo development within a single silique is compromised. Note that the embryo-lethal *icr1* embryos were excluded.
(0.22 MB TIF)

Figure S4 **Abnormal** ***DR5*** **response in** ***icr1*** **embryos.** *DR5rev*::ER-GFP expression in WT and *icr1* embryos. (A–E and L) *Col-0*, (F–K) *icr1*. (A and F) Early globular stage, (B and G) triangular, (C and H) early/mid-heart, and (D, E, and I–L) late heart stage. Panels (K) and (L) are enlargements of the RM of (D) and (I), respectively. Arrows mark the auxin accumulation foci in developing cotyledons. Arrowheads in (C, D, H, I, K, and L) indicate position of QC. Arrowheads in (E and J) point to the downward movement of auxin through provascular tissue. (A–J) Maximum projection Z-stack of multiple confocal sections; (K and L) single confocal scans throughout the center of RM. (A–D, F–I, K, and L) are fluorescence/DIC overlay images. (E) and (J) are fluorescent images. GFP fluorescence is shown in green. Bars correspond to 20 µm.
(2.37 MB TIF)

Figure S5 **Abnormal expression pattern of** ***pWOX5::*** **ER-GFP in** ***icr1*** **embryos.** *WOX5* expression was first detected in globular stage embryos. In WT embryos expression is seen in the lens shape and the upper suspensor cells. In *icr1* globular and heart stage embryos expression is spread to cell neighboring the lens-shape cell (arrowhead). In adult *icr1* embryos strong *WOX5* expression is seen in the lower part of the hypocotyl (arrowhead) and in the cotyledons. In contrast in adult WT embryo *WOX5* expression is detected in the QC and the cotyledons. However, the expression in the cotyledon is weak and its detection required using high detector sensitivity. Bars correspond to 10 µm globular embryos, 20 µm heart stage embryos, and 50 µm adult embryos.
(2.19 MB TIF)

Figure S6 **The expression pattern of** ***SCR*** **is altered in** ***icr1*** **embryos.** Expression pattern of *SCR* was determined with *pSCR::*YFP-His2b reporter seen as yellow nuclei. In WT globular embryos *SCR* is expressed in the hypophysis, ground meristem, and provascular cells. The inset is a projection stack of multiple confocal scans. At the heart stage expression expanded to the QC cells. In adult embryos expression was detected in the QC and future endodermis of the embryonic roots and in the hypocotyls. Note the clear differences in the expression pattern between the hypocotyl and the embryonic root (noted by the rectangular and curved brackets). In bent cotyledon embryos the expression was confined to the QC and endodermis/cortex stem cells of the embryonic root. Low levels of expression were detected in the cotyledons. Abnormal expression pattern of *SCR* was seen in globular stage *icr1* embryos with early developmental aberrations (arrowhead). In heart stage *icr1* embryos expression was spread to additional cells (arrowheads). Similar to WT, in mature *icr1* embryos *SCR* expression was detected in the embryonic root and hypocotyls. However, unlike WT embryos no clear distinction in expression pattern could be made between the embryonic root and the hypocotyls. In bent cotyledons *icr1* embryos *SCR* expression was confined to the embryonic root but was more spread compared to WT embryos (arrowhead). Bars correspond to 10 µm globular embryos, 20 µm heart stage embryos, and 50 µm adult embryos.
(1.78 MB TIF)

Figure S7 **The differentiation of vascular tissues in leaves of** ***icr1*** **is compromised.** Cross-sections across a rosette leave's central vascular strand. The reduced sized of the vascular strand in *icr1* is apparent. Note also the altered mesophyll cell shape and large air spaces in the *icr1* leaf. Bars correspond to 20 µm.
(1.82 MB TIF)

Figure S8 **High-resolution images demonstrating the altered localization of PIN1 and PIN2 in** ***icr1*** **roots.** Arrowheads denote the orientation of PIN localization in cells. Bars are 20 µm.
(1.72 MB TIF)

Figure S9 ***pPIN1*** **-driven GFP-PIN1 localization in WT and** ***icr1*** **roots.** GFP-PIN1 expression in 3 DAG primary root of *Col-0* (A to C) and *icr1* (D to F). (A and D) Overlay between DIC and GFP fluorescence, (B and E) GFP, and (C and F) PIN1-GFP expressing roots stained with 5 µM FM4-64. Insets in (C and F) are close-up views of the RM. (G–I) *pPIN1::*GFP-PIN1 expression in root hairs and epidermis. (G) *Col-0* (H and I) *icr1*. Arrowheads in (H) indicate ectopic expression in epidermis and root hairs. Arrowhead in (I) marks accumulation of GFP-PIN1 in internal bodies. (A, B, D, and E) Maximum projection Z-stack of multiple confocal sections. (C, F, and G–I) single confocal scans. (A, D, G, and H) are fluorescence/DIC overlay images. (B, C, E, F, and I) are fluorescent images. (C and F) are GFP/FM4-64 overlay images. GFP fluorescence is shown in green and FM4-64 in red. Bars correspond to 20 µm (A to F) and 50 µm (G to I).
(2.42 MB TIF)

Figure S10 **Localization of** ***pPIN2*** **-driven GFP-PIN2 in** ***icr1*** **roots.** (A) WT and *icr1* roots expressing *pPIN2*::GFP-PIN2 and stained with FM4-64 at 4 DAG. White boxes indicate the enlarged regions shown on the right panels. Arrows in WT mark apical localization of GFP-PIN2 in the epidermis and basal in the cortex. Note apicalization of GFP-PIN2 in both epidermis and cortex in *icr1* roots (arrows). (B) BFA treatments of WT and *icr1* seedlings resulted in co-localization of FM4-64 and GFP-PIN2 in BFA compartments (arrowheads). Bars correspond to 20 µm.
(3.97 MB TIF)

Figure S11 **The altered** ***pPIN1*** **-driven GFP-PIN1 localization in** ***icr1*** **embryos.** (A–D, I, and J) *Col-0*, (E–H, K, and L) *icr1* embryos showing mild basal defects, (M and N) *icr1* embryos with strong basal defects. (A, B, E, and F) mid-globular stage, (C, D, G, H, M, and N) triangular stage, (I–L) heart stage. Arrowheads in (B, D, F, H, J, and L) indicate the orientation of GFP-PIN1 localization, basal in procambial cells, and apical in protoderm. Arrows in (M and N) indicate the loss of PIN1-GFP expression in the basal region of *icr1* embryos with strong basal defects. Insets in (I and K) are enlargements of a developing cotyledon. (A, C, E, G, I, K, and M) Maximum projection Z-stack of multiple confocal sections. (B, D, F, H, J, and N) Single confocal scans throughout the center of embryo. (A, C, E, G, I, M, and K) are fluorescence/DIC overlay images. (B, D, F, H, J, L, N, and insert in I and K) are fluorescent images. GFP fluorescence is shown in green. Bars correspond to 20 µm.
(2.87 MB TIF)

Figure S12 **A model summarizing PIN1 localization, auxin distribution, and patterning in WT and** ***icr1*** **embryos.** Cell outlines of WT (A) and *icr1* embryos (B and C). (A) Polar membrane localization of PIN1 mediates directional auxin flux and appearance of auxin maxima in embryonic root meristem and future cotyledon tips during the development. (B) Reduced PIN1 polarity results in weak auxin flux and gradually disrupts formation of auxin maxima in *icr1* embryos with late patterning defects. (C) In *icr1* embryos with early patterning defects PIN1 membrane localization and polarity are strongly affected, likely leading to non-polar auxin distribution (crossed arrows).
(1.24 MB TIF)

Figure S13 **The** ***pICR1::LhG4*** **and the** ***pOp::ICR1_5′-UTR_-GFP-ICR1_genomic_*** **constructs.** (A) The *pICR1* driven effector/reporter lines. The *LhG4/pOp* system allows expression of different reporters from the same effector, thereby reducing positional effects on gene expression [35],[36]. In this work, GFP-ICR1, GFP-rop6^CA^, and GUS were expressed using the same pICR1 effector lines. (B) Schematic representations of the *pICR1* promoter. (C) The GFP-ICR_genomic_ construct.
(0.30 MB TIF)

Figure S14 **ICR1 expression is induced by auxin.** *pICR1*≫GUS expression following growth on NPA or induction by NAA for 6 h. Arrows denote GUS expression in pericycle cells following NAA treatments. Bars correspond to 50 µm.
(2.96 MB TIF)

Figure S15 **Complementation of root growth in** ***icr1*** **mutants by GFP-ICR1.** Expression of GFP-ICR1 was driven by the ICR1 promoter using transcription/transactivation system (see Figure S12). Bars correspond to 100 µm.
(0.61 MB TIF)

Figure S16 **Plasma membrane localization of GFP-ICR1 detected following plasmolysis.** PI-stained GFP-ICR1 expressing leaf epidermis pavement cells before and after plasmolysis. The bottom large panel is a magnification of the overlay panel after plasmolysis. Red arrowheads denote the cell wall and the white arrowheads denote the detached plasma membrane. The fluorescent green patches detected after plasmolysis indicate that some of the GFP-ICR1 was not attached to the plasma membrane. Bars correspond to 20 µm.
(5.46 MB TIF)

Figure S17 **Effect of the apoplastic pH on secGFP fluorescence and its localization in WT and** ***icr1*** **roots.** (A) Mean fluorescence of WT and *icr1* roots at 5 DAG that were transferred and incubated for 3 h in liquid MS medium titrated to either pH 5.5 or pH 8.5. Error bars correspond to SE, *n*≥20, ** significant differences in fluorescence of WT roots were detected between pH 8.5 and 5.5 (*p*≤0.01; Student's *t* test). Fluorescence differences in *icr1* between pH 8.5 and 5.5 were insignificant (*p*≥0.72). (B) A WT root incubated in MS medium titrated to pH 8.5 stained with PI (red). Arrows denote the apoplastic localization of secGFP. (C) An *icr1* root incubated in MS medium tittered to pH 8.5 and stained with PI (red). Note that the GFP fluorescence remained intracellular. Bars correspond to 10 µm.
(0.46 MB TIF)

Table S1 **The frequency of** ***icr1*** **^−/−^ embryos exhibiting patterning defects at indicated developmental stages.** * Embryos with strong basal defects were excluded from analysis. **^1^** Embryos were analyzed at 1-cell, 2-cell, 4-cell, 8-cell (octant), and 16-cell (dermatogen) stages.
(0.04 MB DOC)

Video S1 **Expression of** ***pICR1*** **≫GFP-ICR1 at the root tip.** The movie highlights the absence of ICR1 expression at and around the QC.
(10.12 MB AVI)

## Abbreviations

ARF: ADP ribosylation factor
BFA: brefeldin A
bp: base pair
CA: Constitutive Active
CLSM: Confocal Laser Scanning Microscope
Col-0: Columbia-0
DAG: days after germination
DIC: Differential Interference Contrast
FRAP: Fluorescence Recovery After Photobleaching
GEF: Guanine Nucleotide Exchange Factor
GFP: Green Fluorescent Protein
GUS: βGlucoronidase
HLR: HALTEDROOT
ICR1: Interactor of Constitutive active ROP 1
MS: Murashige & Skoog
MT: microtubules
NAA: Naphthalene Acetic Acid
NPA: 1-N-naphthylphthalamic acid
PI: Propidium Iodide
PID: PINOID
PIN: PINFORMED
PtdIns P-K: phosphatidylinositol monophosphate kinase
QC: quiescent center
q-PCR: Quantitative Real-Time RT-PCR
RIP1: ROP Interacting Protein 1
ROP: Rho of Plants
ROS: Reactive Oxygen Species
SCR: SCARECRAW
SHR: SHORTROOT
UTR: Untranslated Region
WOX5: WUSCHEL related homeobox 5
WT: wild type
