# Supplementary figures and images for: A Rho Scaffold Integrates the Secretory System with Feedback Mechanisms in Regulation of Auxin Distribution
Source: PLoS Biol. 2010 Jan 19;8(1):e1000282. doi: 10.1371/journal.pbio.1000282 (PMC2808208; doi:10.1371/journal.pbio.1000282)

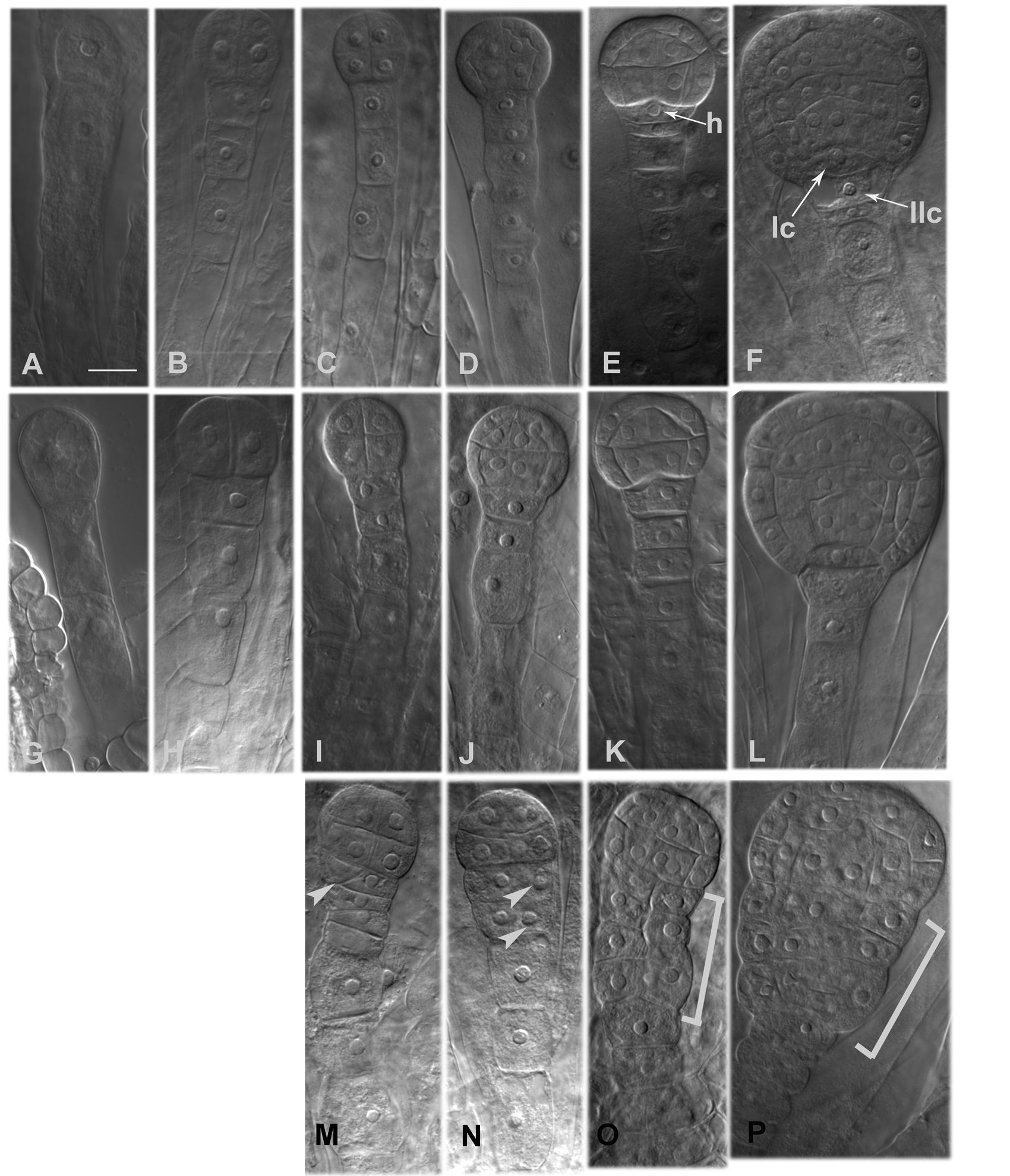

Supplement: Figure S1 — Abnormal development of early icr1 embryos. (A–F) Col-0, (G–L) 90% of icr1 progeny with normal development at early embryo development, (M–P) 10% of icr1 progeny with early basal defects. (A and G) 1-cell stage, (B and H) 4-cell stage, (C, I, and M) 8-cell stage, (D, J, and N) 16-cell stage, (E, K, and O) early globular stage, and (F, L, and P) late globular. h, hypophysis; lc, lens-shaped cell; llc, large lower cell. Arrowheads in (M and N) mark abnormal division in hypophysis and suspensor; vertical brackets in (O and P) mark unshaped basal region. Bars correspond to 10 µm for all images. (1.88 MB TIF) [file pbio.1000282.s001.tif]

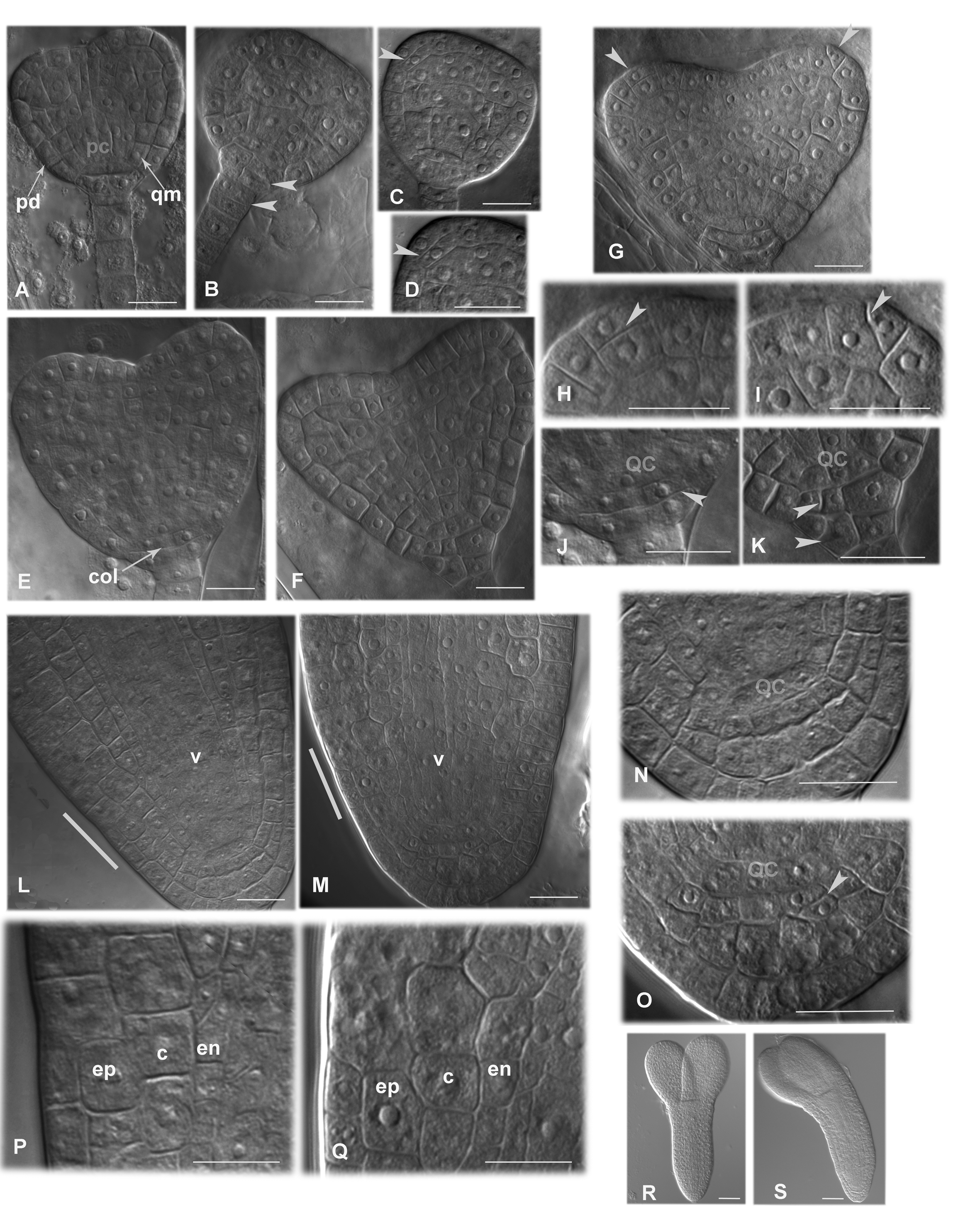

Supplement: Figure S2 — Abnormal development of late icr1 embryos. (A–D) Triangular stage, (A) Col-0, and (B–D) icr1. Arrowheads in (B) indicate abnormal divisions in suspensor and columella initials; arrowhead in (C and D) marks abnormal division in protoderm. (D) Enlargement of the arrow-highlighted section in (C). (E–K) Early/mid-heart stage, (E and J) Col-0, (F–I and K) icr1. Arrowheads in (G–I) mark abnormal divisions in protoderm. Arrowhead in (J) indicates a normal periclinal division in a WT columella. Arrowheads in (K) point to abnormal division planes in columella initials of icr1 embryos. (L–S) A bent cotyledon stage, (L, N, P, and R) Col-0, and (M, O, Q, and S) icr1. (N and O) Enlargement of the root meristem (RM). The arrowhead in (O) marks abnormal division in columella. Vertical bars in (L and M) indicate enlarged region shown in (P and Q). pd, protoderm; pc, procambium; col, columella initials; QC, quiescent center; v, vascular tissue; ep, epidermis; c, cortex; en, endodermis. Bars correspond to 20 µm (A–M), 10 µm (N–Q), and 50 µm (R and S). (3.72 MB TIF) [file pbio.1000282.s002.tif]

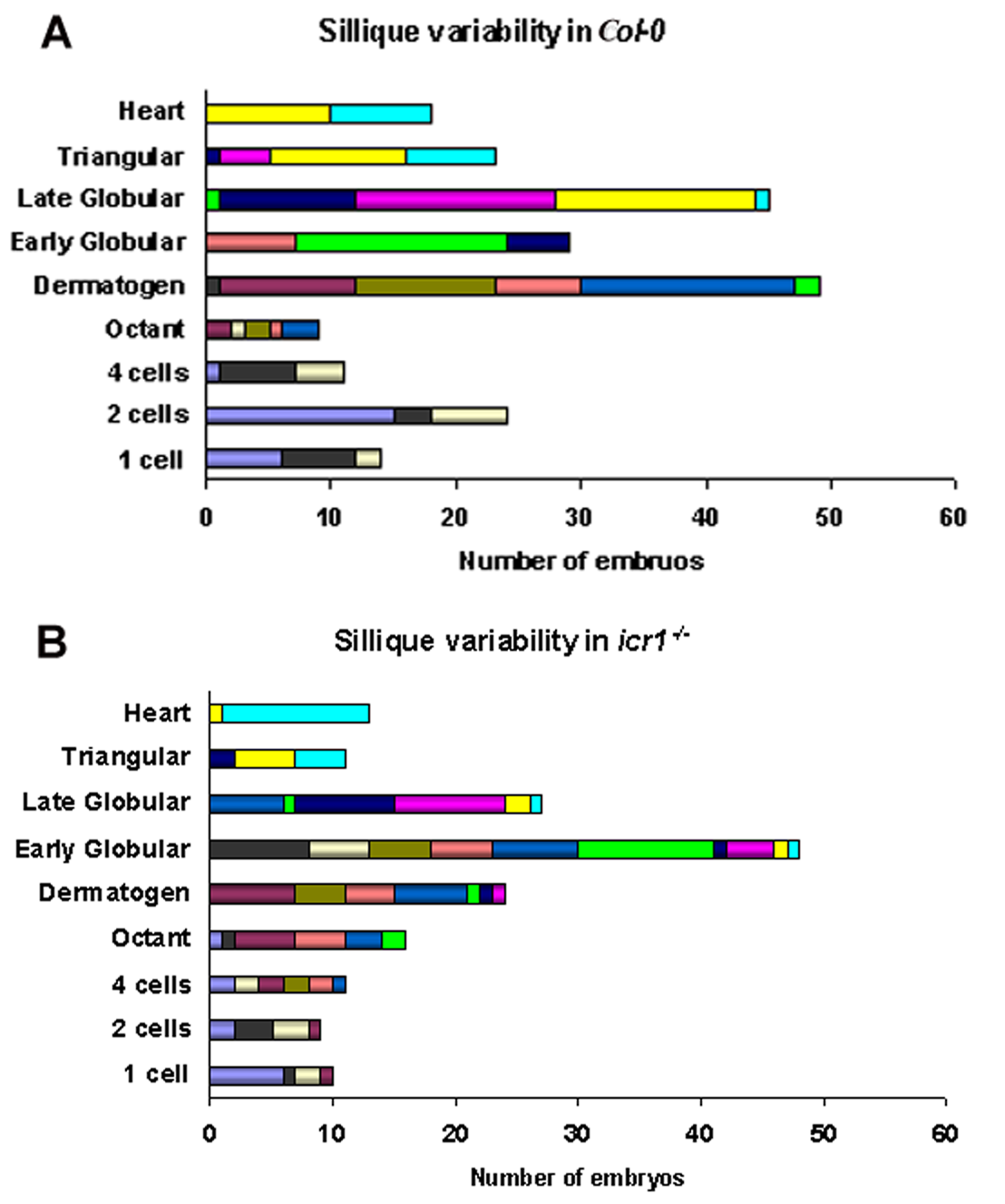

Supplement: Figure S3 — icr1 siliques display greater developmental variability. Stacked-bar charts show the developmental stages of embryos collected from 12 representative siliques. (A) Col-0 and (B) icr1 siliques of various ages. Each color marks a single silique. The high mixing of colors in icr1 mutant indicates that the synchronization of embryo development within a single silique is compromised. Note that the embryo-lethal icr1 embryos were excluded. (0.22 MB TIF) [file pbio.1000282.s003.tif]

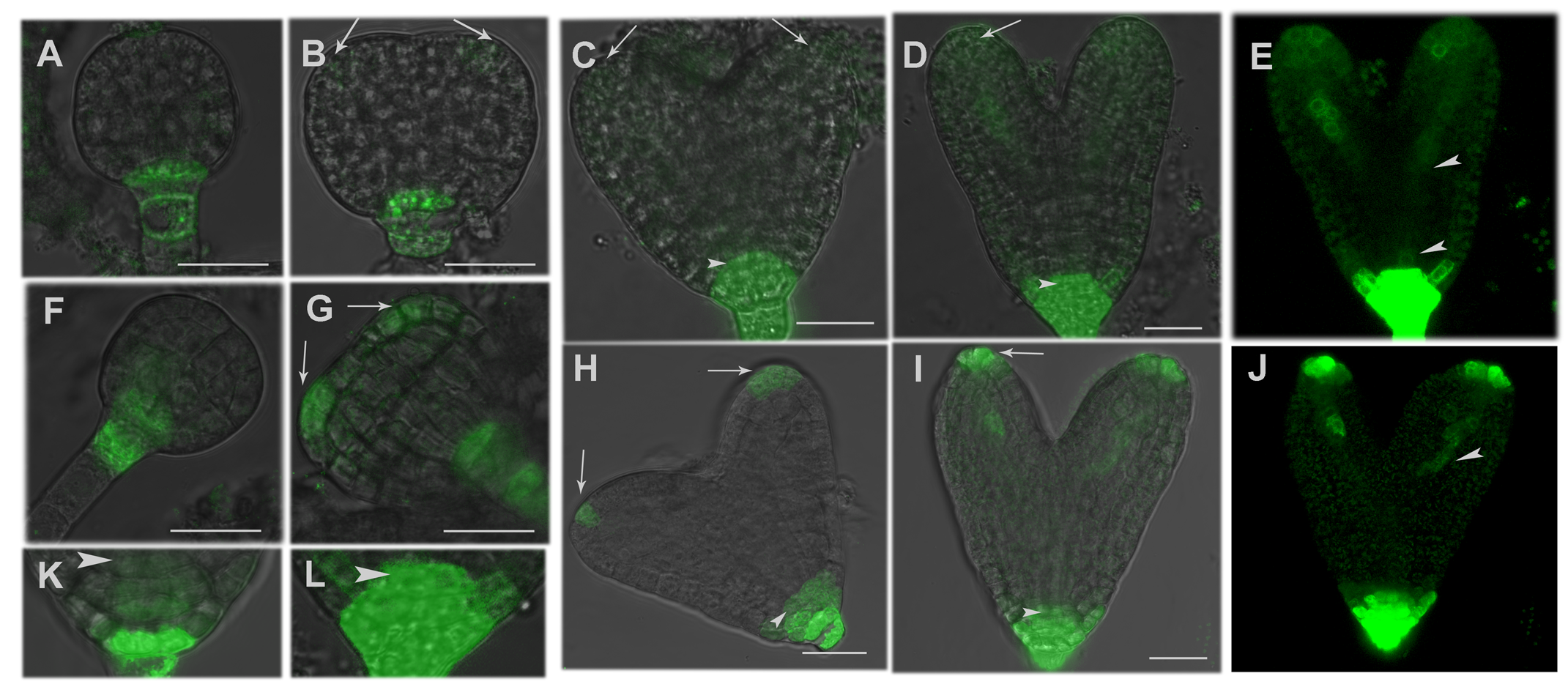

Supplement: Figure S4 — Abnormal DR5 response in icr1 embryos. DR5rev::ER-GFP expression in WT and icr1 embryos. (A–E and L) Col-0, (F–K) icr1. (A and F) Early globular stage, (B and G) triangular, (C and H) early/mid-heart, and (D, E, and I–L) late heart stage. Panels (K) and (L) are enlargements of the RM of (D) and (I), respectively. Arrows mark the auxin accumulation foci in developing cotyledons. Arrowheads in (C, D, H, I, K, and L) indicate position of QC. Arrowheads in (E and J) point to the downward movement of auxin through provascular tissue. (A–J) Maximum projection Z-stack of multiple confocal sections; (K and L) single confocal scans throughout the center of RM. (A–D, F–I, K, and L) are fluorescence/DIC overlay images. (E) and (J) are fluorescent images. GFP fluorescence is shown in green. Bars correspond to 20 µm. (2.37 MB TIF) [file pbio.1000282.s004.tif]

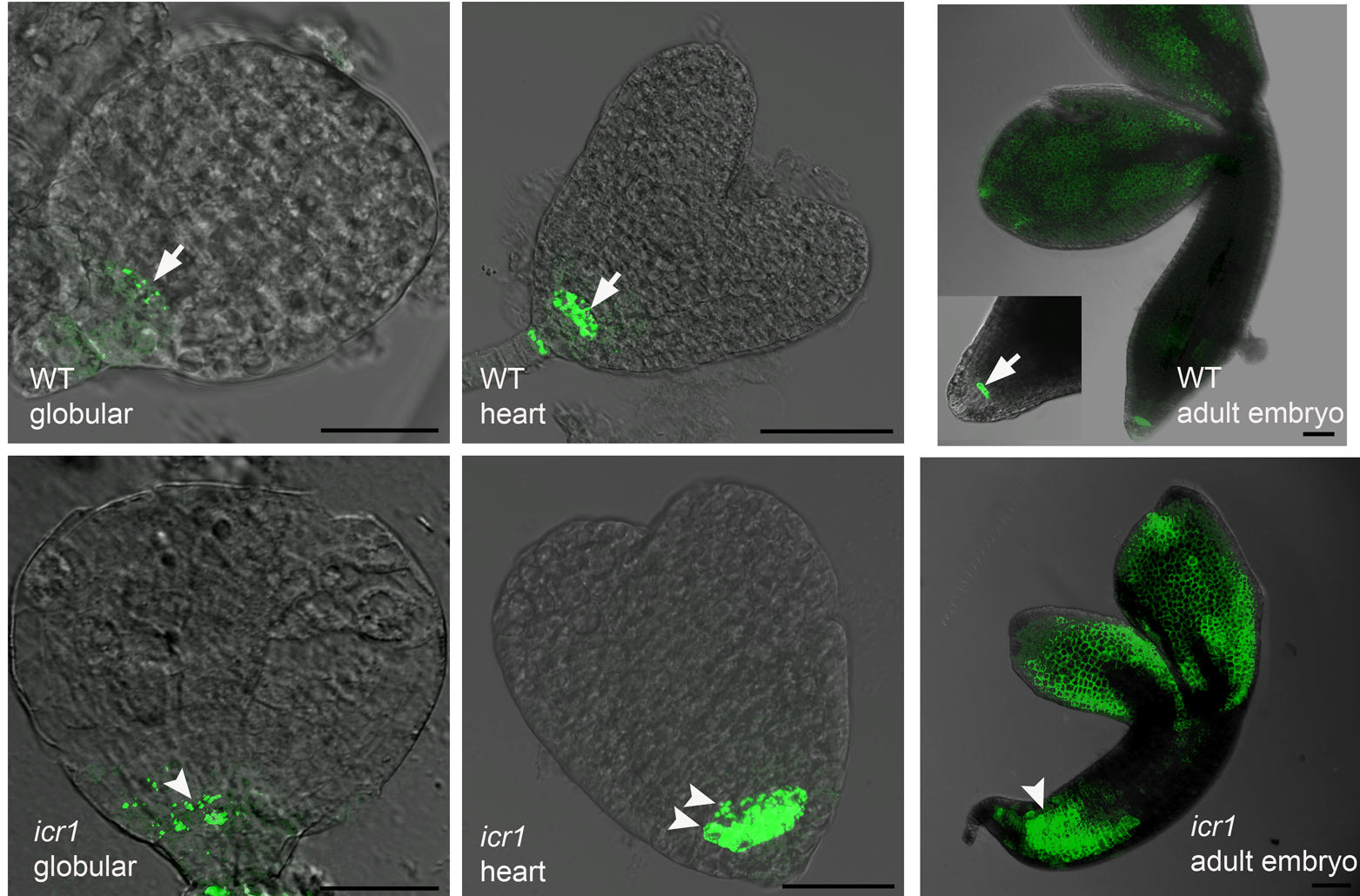

Supplement: Figure S5 — Abnormal expression pattern of pWOX5:: ER-GFP in icr1 embryos. WOX5 expression was first detected in globular stage embryos. In WT embryos expression is seen in the lens shape and the upper suspensor cells. In icr1 globular and heart stage embryos expression is spread to cell neighboring the lens-shape cell (arrowhead). In adult icr1 embryos strong WOX5 expression is seen in the lower part of the hypocotyl (arrowhead) and in the cotyledons. In contrast in adult WT embryo WOX5 expression is detected in the QC and the cotyledons. However, the expression in the cotyledon is weak and its detection required using high detector sensitivity. Bars correspond to 10 µm globular embryos, 20 µm heart stage embryos, and 50 µm adult embryos. (2.19 MB TIF) [file pbio.1000282.s005.tif]

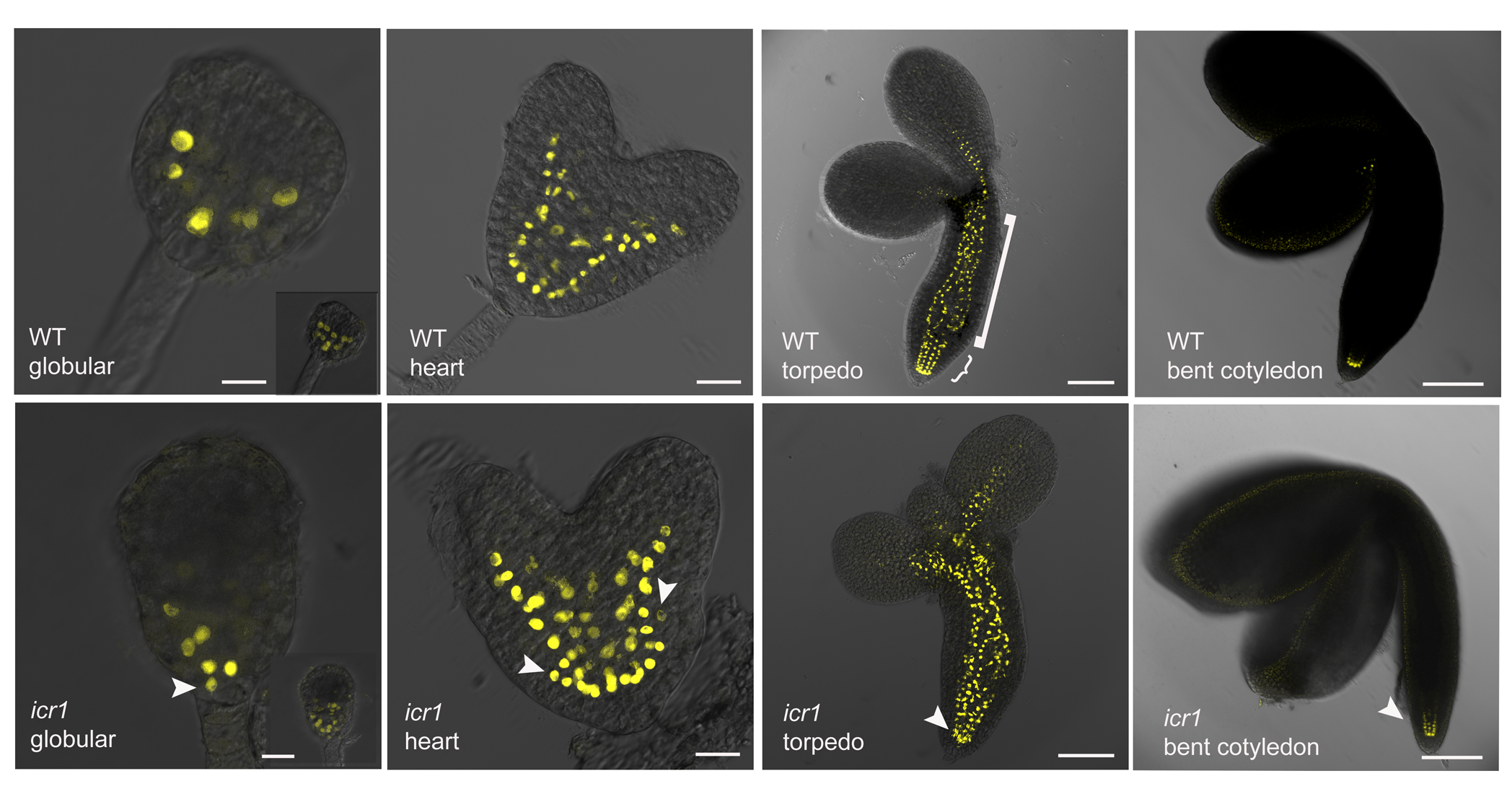

Supplement: Figure S6 — The expression pattern of SCR is altered in icr1 embryos. Expression pattern of SCR was determined with pSCR::YFP-His2b reporter seen as yellow nuclei. In WT globular embryos SCR is expressed in the hypophysis, ground meristem, and provascular cells. The inset is a projection stack of multiple confocal scans. At the heart stage expression expanded to the QC cells. In adult embryos expression was detected in the QC and future endodermis of the embryonic roots and in the hypocotyls. Note the clear differences in the expression pattern between the hypocotyl and the embryonic root (noted by the rectangular and curved brackets). In bent cotyledon embryos the expression was confined to the QC and endodermis/cortex stem cells of the embryonic root. Low levels of expression were detected in the cotyledons. Abnormal expression pattern of SCR was seen in globular stage icr1 embryos with early developmental aberrations (arrowhead). In heart stage icr1 embryos expression was spread to additional cells (arrowheads). Similar to WT, in mature icr1 embryos SCR expression was detected in the embryonic root and hypocotyls. However, unlike WT embryos no clear distinction in expression pattern could be made between the embryonic root and the hypocotyls. In bent cotyledons icr1 embryos SCR expression was confined to the embryonic root but was more spread compared to WT embryos (arrowhead). Bars correspond to 10 µm globular embryos, 20 µm heart stage embryos, and 50 µm adult embryos. (1.78 MB TIF) [file pbio.1000282.s006.tif]

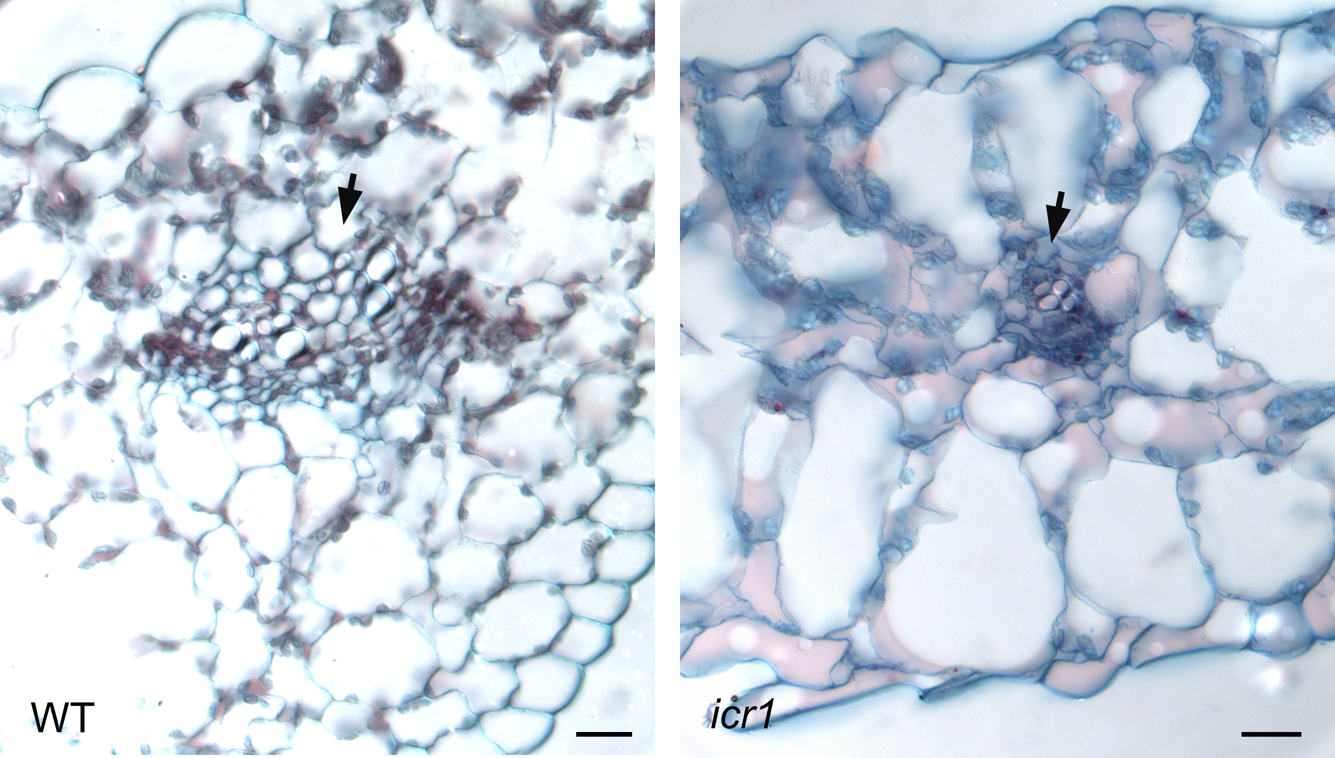

Supplement: Figure S7 — The differentiation of vascular tissues in leaves of icr1 is compromised. Cross-sections across a rosette leave's central vascular strand. The reduced sized of the vascular strand in icr1 is apparent. Note also the altered mesophyll cell shape and large air spaces in the icr1 leaf. Bars correspond to 20 µm. (1.82 MB TIF) [file pbio.1000282.s007.tif]

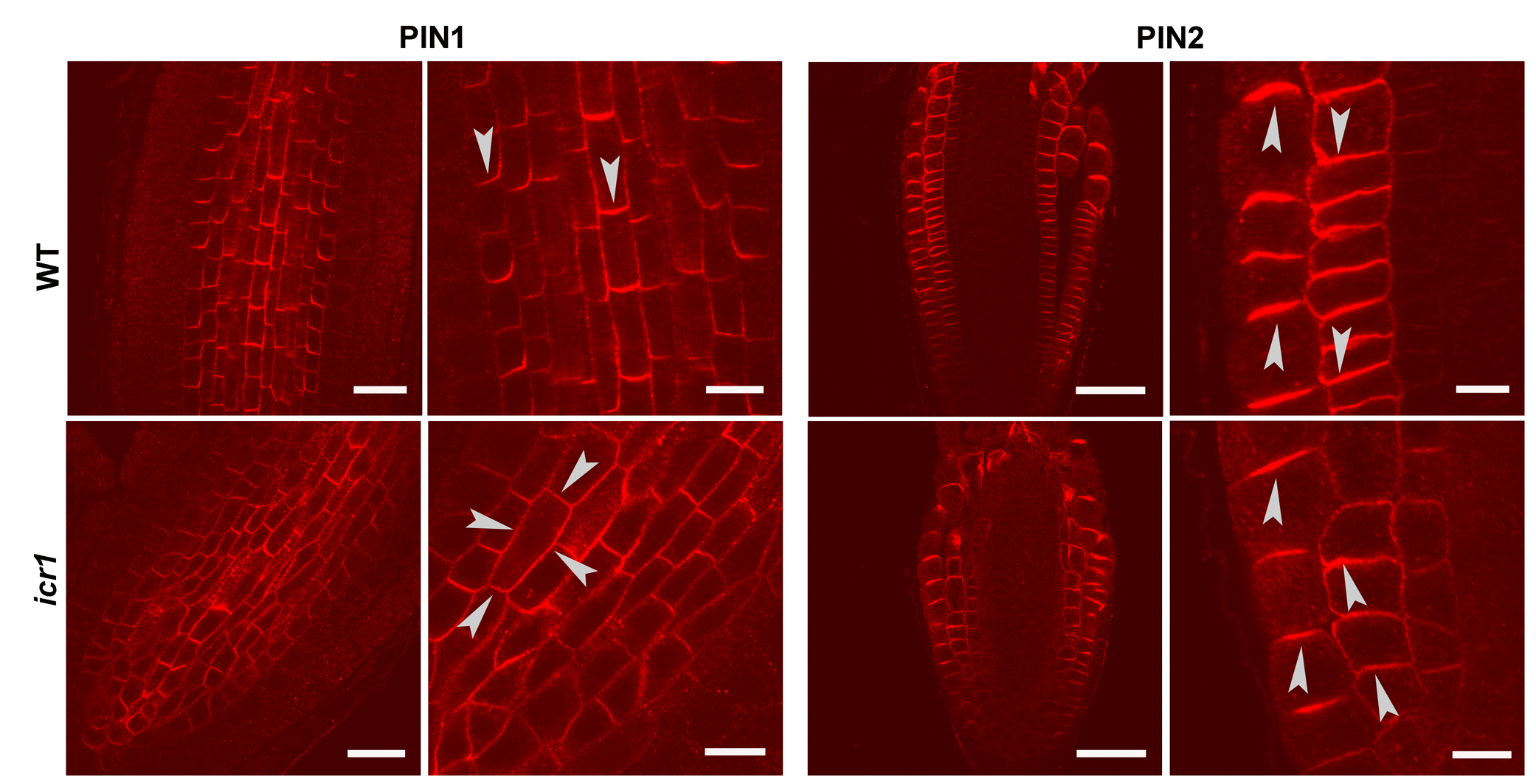

Supplement: Figure S8 — High-resolution images demonstrating the altered localization of PIN1 and PIN2 in icr1 roots. Arrowheads denote the orientation of PIN localization in cells. Bars are 20 µm. (1.72 MB TIF) [file pbio.1000282.s008.tif]

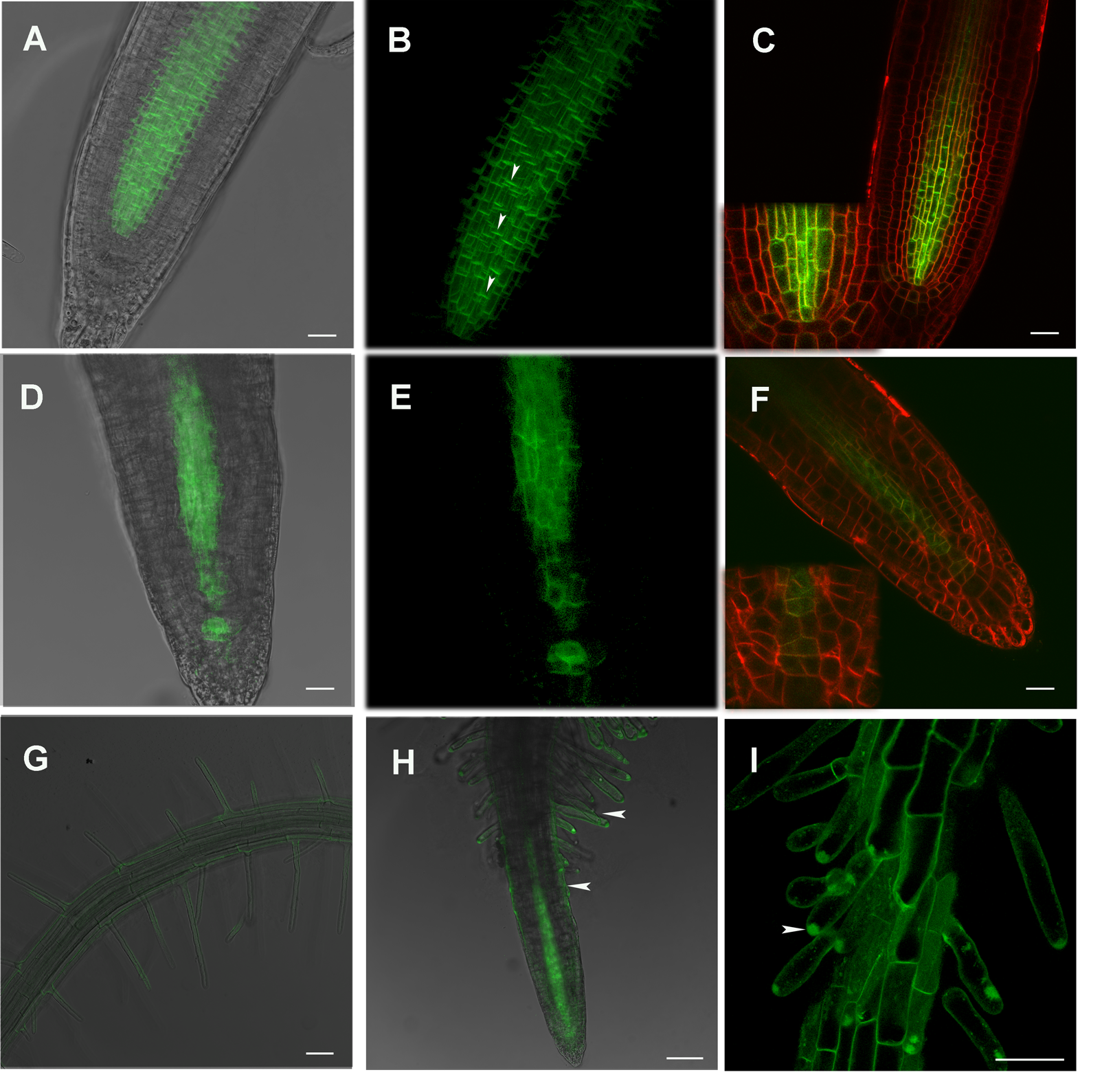

Supplement: Figure S9 — pPIN1 -driven GFP-PIN1 localization in WT and icr1 roots. GFP-PIN1 expression in 3 DAG primary root of Col-0 (A to C) and icr1 (D to F). (A and D) Overlay between DIC and GFP fluorescence, (B and E) GFP, and (C and F) PIN1-GFP expressing roots stained with 5 µM FM4-64. Insets in (C and F) are close-up views of the RM. (G–I) pPIN1::GFP-PIN1 expression in root hairs and epidermis. (G) Col-0 (H and I) icr1. Arrowheads in (H) indicate ectopic expression in epidermis and root hairs. Arrowhead in (I) marks accumulation of GFP-PIN1 in internal bodies. (A, B, D, and E) Maximum projection Z-stack of multiple confocal sections. (C, F, and G–I) single confocal scans. (A, D, G, and H) are fluorescence/DIC overlay images. (B, C, E, F, and I) are fluorescent images. (C and F) are GFP/FM4-64 overlay images. GFP fluorescence is shown in green and FM4-64 in red. Bars correspond to 20 µm (A to F) and 50 µm (G to I). (2.42 MB TIF) [file pbio.1000282.s009.tif]

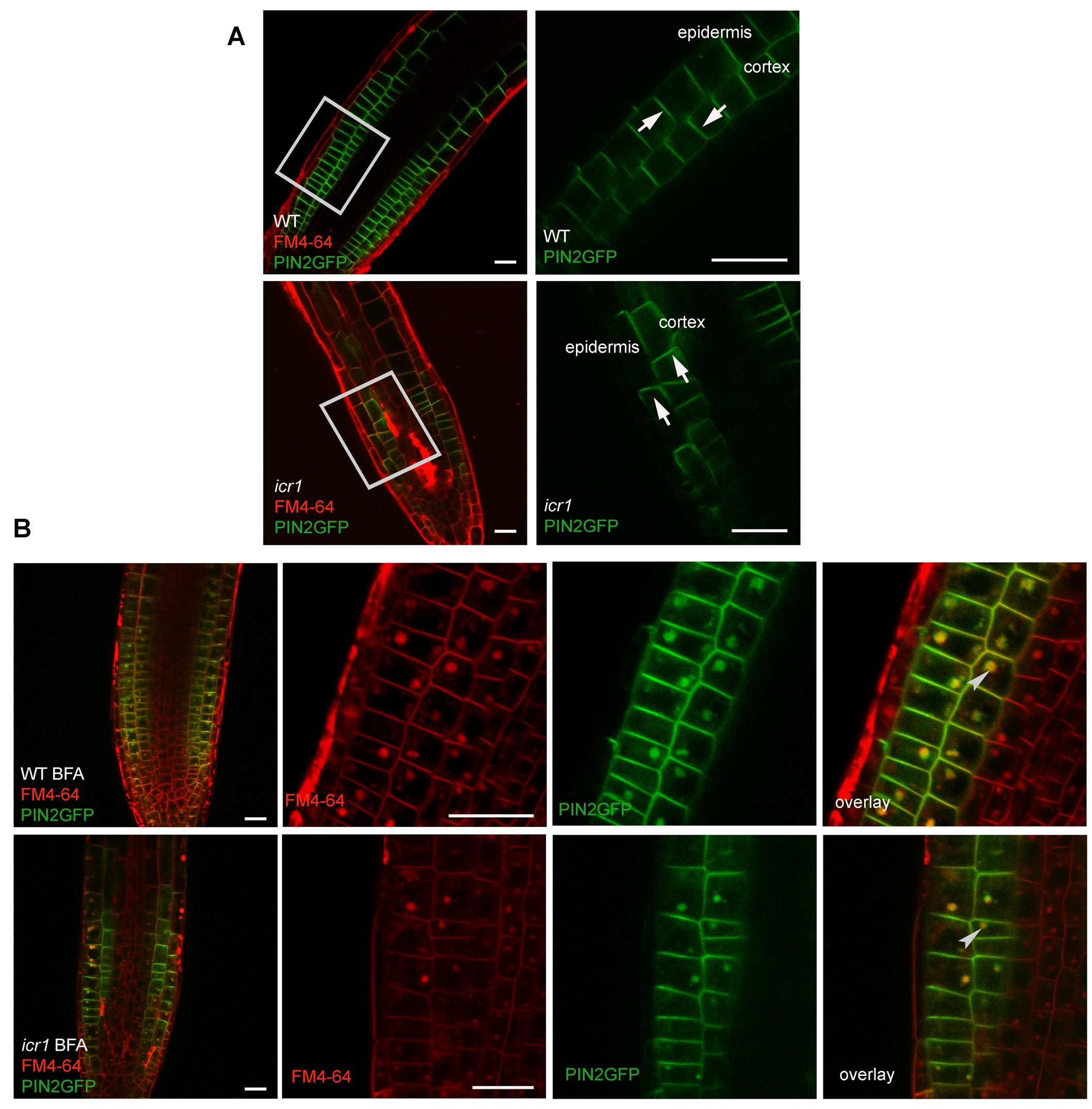

Supplement: Figure S10 — Localization of pPIN2 -driven GFP-PIN2 in icr1 roots. (A) WT and icr1 roots expressing pPIN2::GFP-PIN2 and stained with FM4-64 at 4 DAG. White boxes indicate the enlarged regions shown on the right panels. Arrows in WT mark apical localization of GFP-PIN2 in the epidermis and basal in the cortex. Note apicalization of GFP-PIN2 in both epidermis and cortex in icr1 roots (arrows). (B) BFA treatments of WT and icr1 seedlings resulted in co-localization of FM4-64 and GFP-PIN2 in BFA compartments (arrowheads). Bars correspond to 20 µm. (3.97 MB TIF) [file pbio.1000282.s010.tif]

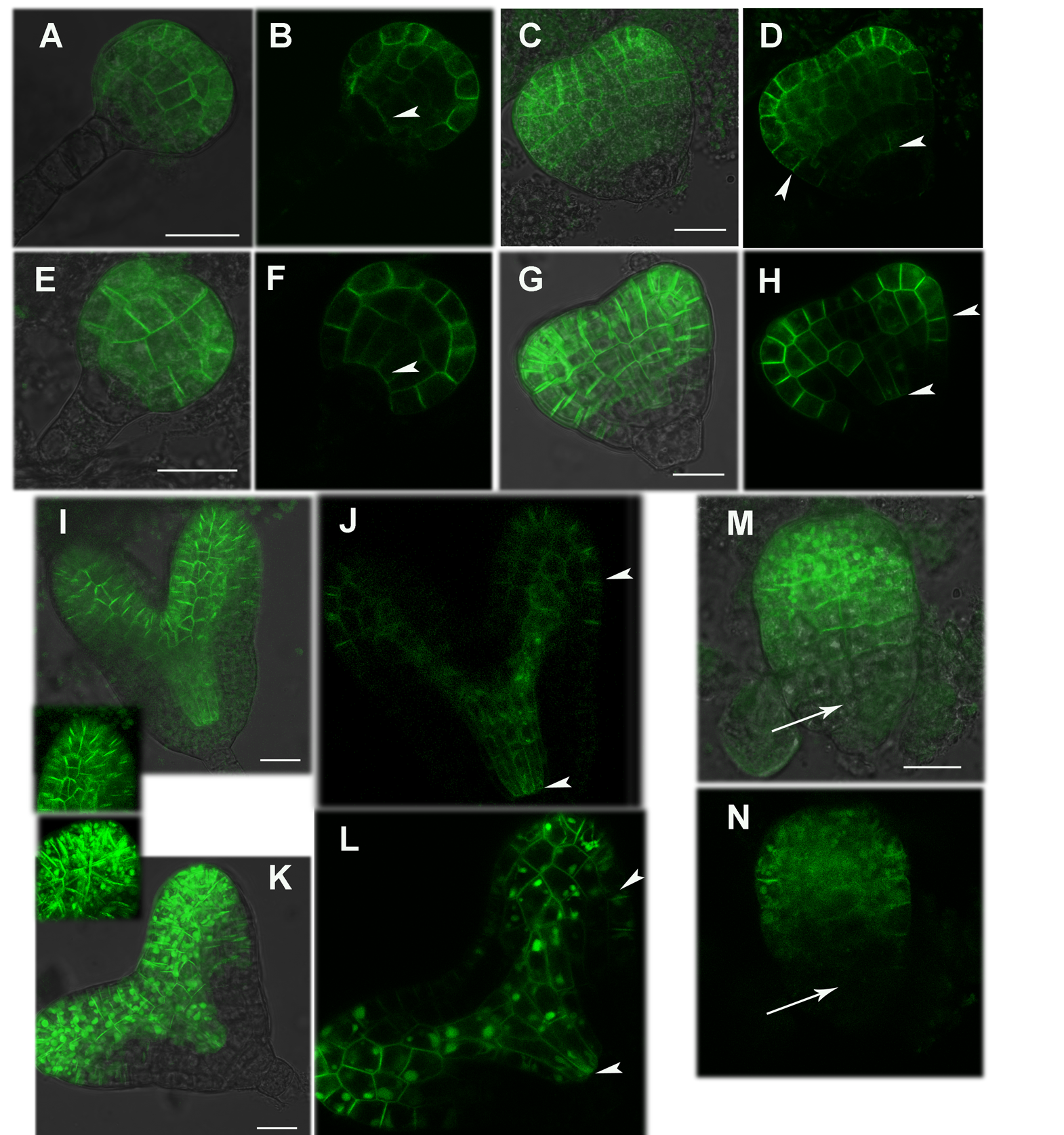

Supplement: Figure S11 — The altered pPIN1 -driven GFP-PIN1 localization in icr1 embryos. (A–D, I, and J) Col-0, (E–H, K, and L) icr1 embryos showing mild basal defects, (M and N) icr1 embryos with strong basal defects. (A, B, E, and F) mid-globular stage, (C, D, G, H, M, and N) triangular stage, (I–L) heart stage. Arrowheads in (B, D, F, H, J, and L) indicate the orientation of GFP-PIN1 localization, basal in procambial cells, and apical in protoderm. Arrows in (M and N) indicate the loss of PIN1-GFP expression in the basal region of icr1 embryos with strong basal defects. Insets in (I and K) are enlargements of a developing cotyledon. (A, C, E, G, I, K, and M) Maximum projection Z-stack of multiple confocal sections. (B, D, F, H, J, and N) Single confocal scans throughout the center of embryo. (A, C, E, G, I, M, and K) are fluorescence/DIC overlay images. (B, D, F, H, J, L, N, and insert in I and K) are fluorescent images. GFP fluorescence is shown in green. Bars correspond to 20 µm. (2.87 MB TIF) [file pbio.1000282.s011.tif]

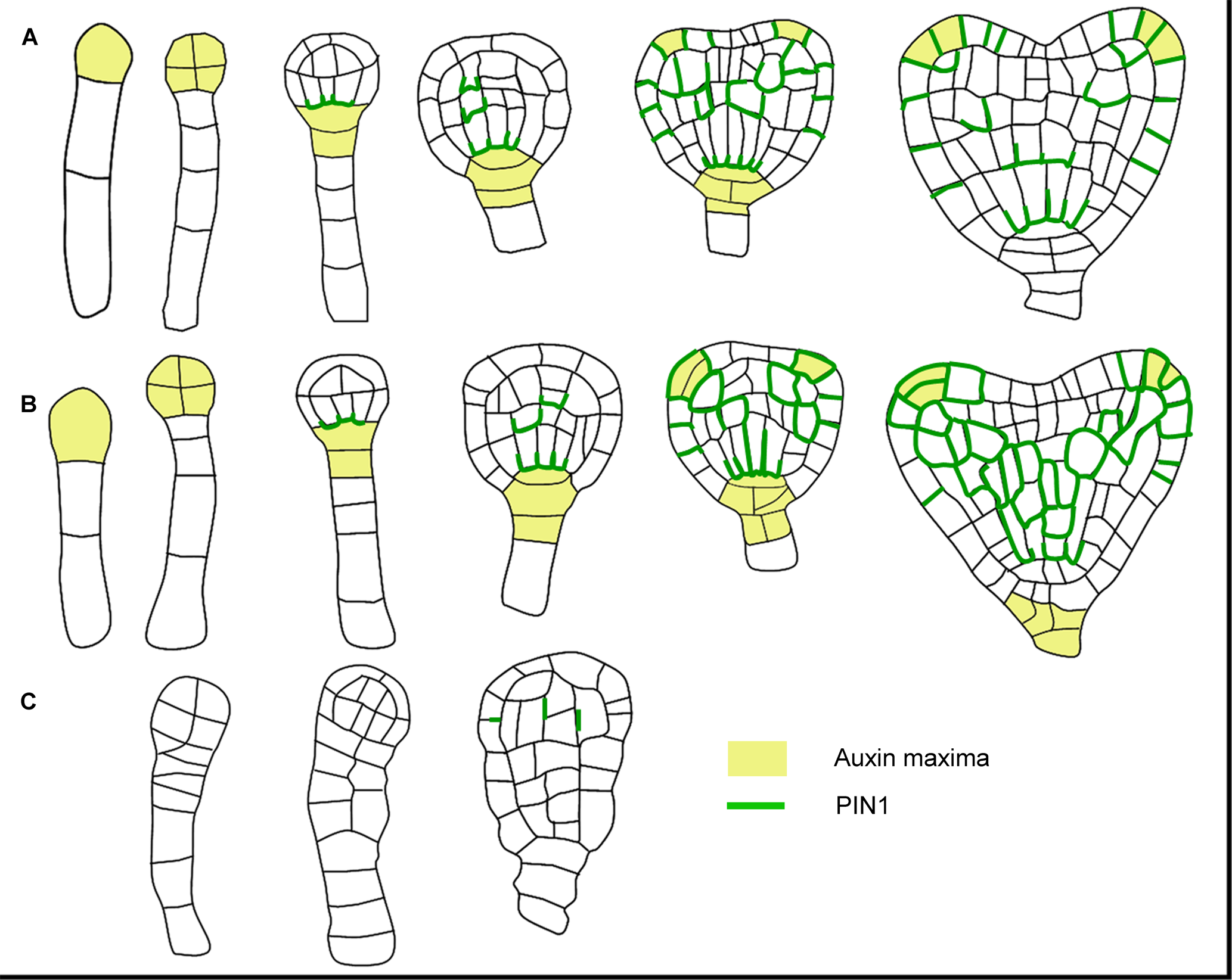

Supplement: Figure S12 — A model summarizing PIN1 localization, auxin distribution, and patterning in WT and icr1 embryos. Cell outlines of WT (A) and icr1 embryos (B and C). (A) Polar membrane localization of PIN1 mediates directional auxin flux and appearance of auxin maxima in embryonic root meristem and future cotyledon tips during the development. (B) Reduced PIN1 polarity results in weak auxin flux and gradually disrupts formation of auxin maxima in icr1 embryos with late patterning defects. (C) In icr1 embryos with early patterning defects PIN1 membrane localization and polarity are strongly affected, likely leading to non-polar auxin distribution (crossed arrows). (1.24 MB TIF) [file pbio.1000282.s012.tif]

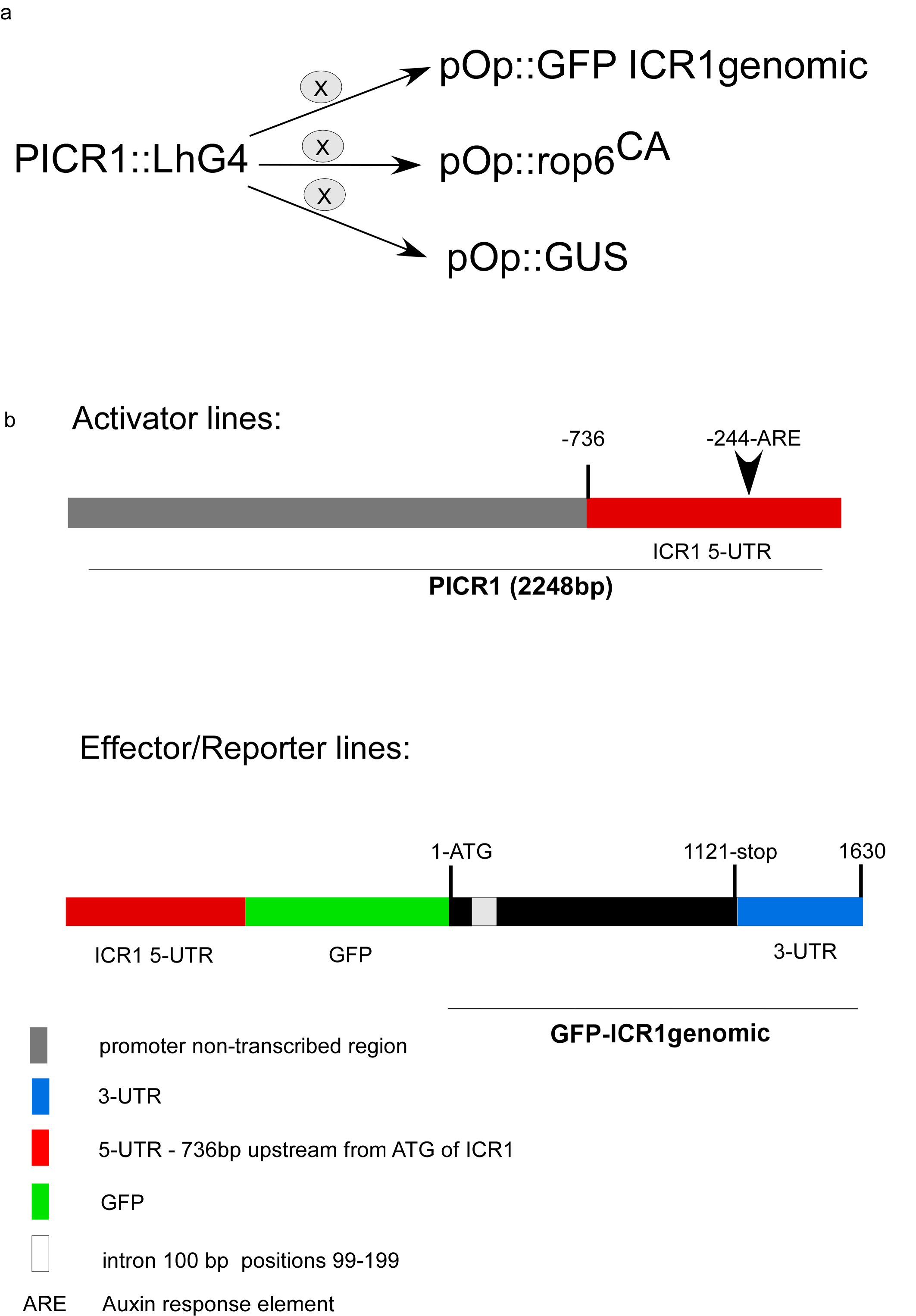

Supplement: Figure S13 — The pICR1::LhG4 and the pOp::ICR15′-UTR-GFP-ICR1genomic constructs. (A) The pICR1 driven effector/reporter lines. The LhG4/pOp system allows expression of different reporters from the same effector, thereby reducing positional effects on gene expression [35],[36]. In this work, GFP-ICR1, GFP-rop6CA, and GUS were expressed using the same pICR1 effector lines. (B) Schematic representations of the pICR1 promoter. (C) The GFP-ICRgenomic construct. (0.30 MB TIF) [file pbio.1000282.s013.tif]

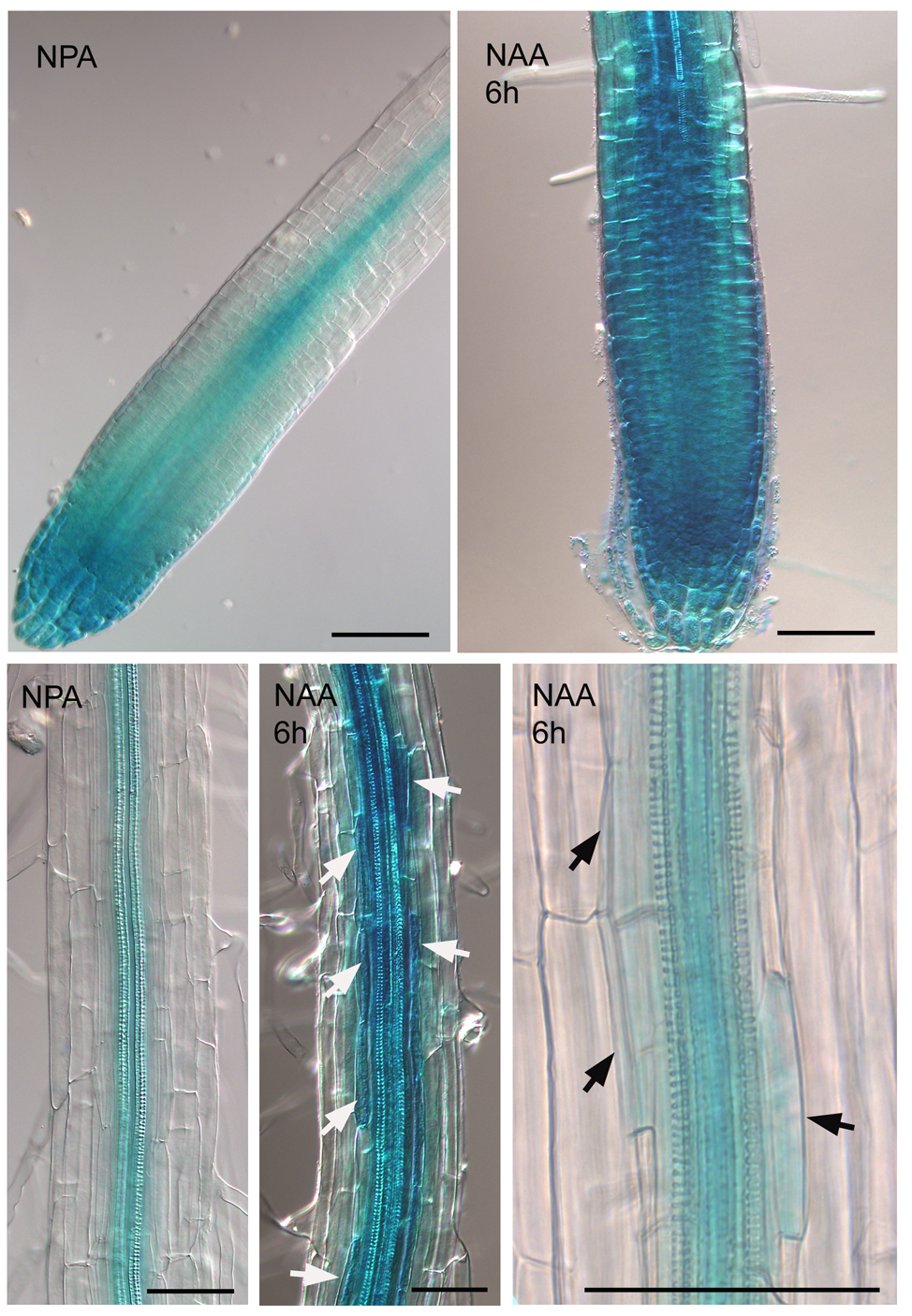

Supplement: Figure S14 — ICR1 expression is induced by auxin. pICR1≫GUS expression following growth on NPA or induction by NAA for 6 h. Arrows denote GUS expression in pericycle cells following NAA treatments. Bars correspond to 50 µm. (2.96 MB TIF) [file pbio.1000282.s014.tif]

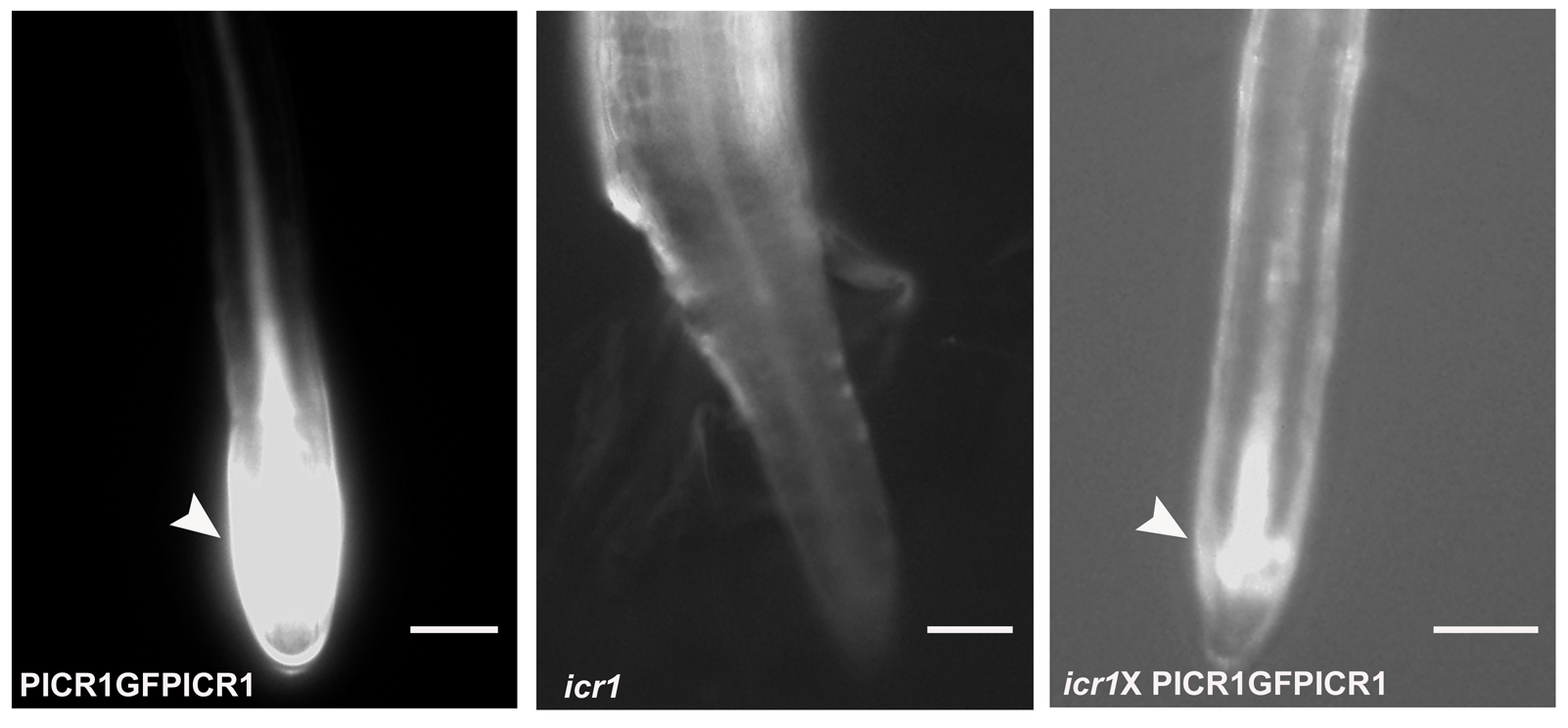

Supplement: Figure S15 — Complementation of root growth in icr1 mutants by GFP-ICR1. Expression of GFP-ICR1 was driven by the ICR1 promoter using transcription/transactivation system (see Figure S12). Bars correspond to 100 µm. (0.61 MB TIF) [file pbio.1000282.s015.tif]

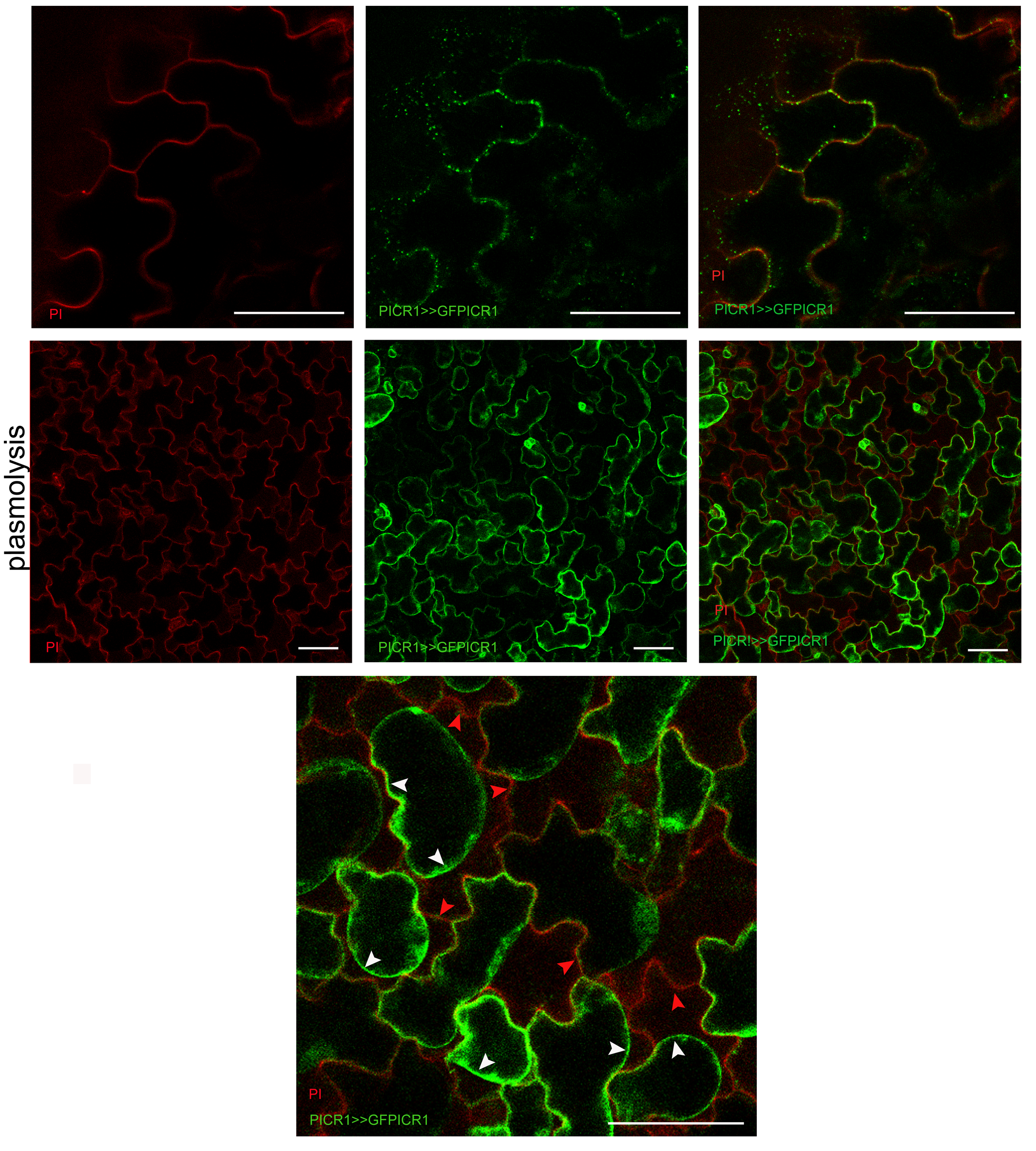

Supplement: Figure S16 — Plasma membrane localization of GFP-ICR1 detected following plasmolysis. PI-stained GFP-ICR1 expressing leaf epidermis pavement cells before and after plasmolysis. The bottom large panel is a magnification of the overlay panel after plasmolysis. Red arrowheads denote the cell wall and the white arrowheads denote the detached plasma membrane. The fluorescent green patches detected after plasmolysis indicate that some of the GFP-ICR1 was not attached to the plasma membrane. Bars correspond to 20 µm. (5.46 MB TIF) [file pbio.1000282.s016.tif]

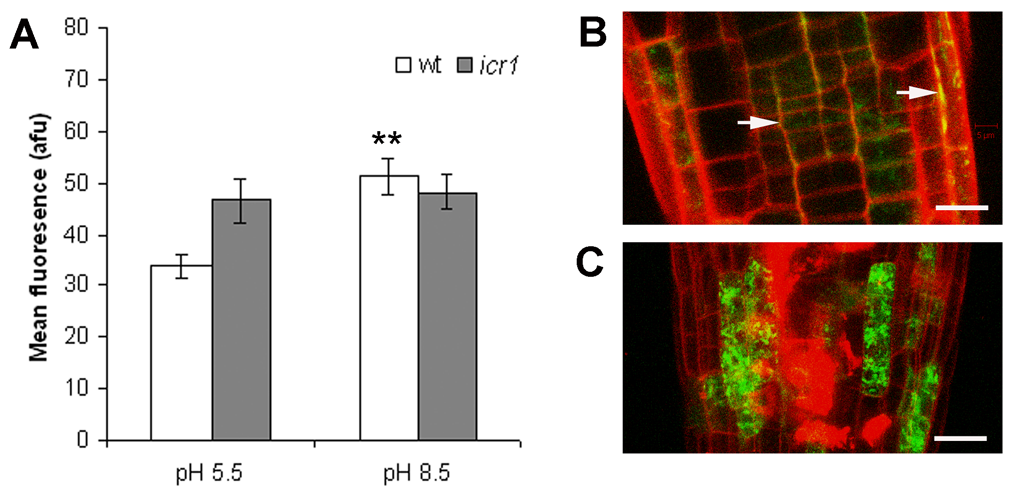

Supplement: Figure S17 — Effect of the apoplastic pH on secGFP fluorescence and its localization in WT and icr1 roots. (A) Mean fluorescence of WT and icr1 roots at 5 DAG that were transferred and incubated for 3 h in liquid MS medium titrated to either pH 5.5 or pH 8.5. Error bars correspond to SE, n≥20, ** significant differences in fluorescence of WT roots were detected between pH 8.5 and 5.5 (p≤0.01; Student's t test). Fluorescence differences in icr1 between pH 8.5 and 5.5 were insignificant (p≥0.72). (B) A WT root incubated in MS medium titrated to pH 8.5 stained with PI (red). Arrows denote the apoplastic localization of secGFP. (C) An icr1 root incubated in MS medium tittered to pH 8.5 and stained with PI (red). Note that the GFP fluorescence remained intracellular. Bars correspond to 10 µm. (0.46 MB TIF) [file pbio.1000282.s017.tif]
