# Supplementary material for: A Rho Scaffold Integrates the Secretory System with Feedback Mechanisms in Regulation of Auxin Distribution
Source: PLoS Biol. 2010 Jan 19;8(1):e1000282. doi: 10.1371/journal.pbio.1000282 (PMC2808208; doi:10.1371/journal.pbio.1000282)
Supplement: Table S1 — The frequency of icr1 −/− embryos exhibiting patterning defects at indicated developmental stages. * Embryos with strong basal defects were excluded from analysis. 1 Embryos were analyzed at 1-cell, 2-cell, 4-cell, 8-cell (octant), and 16-cell (dermatogen) stages. (0.04 MB DOC) [file pbio.1000282.s018.doc]

**Tabel S1.**

|  | **Proembryo^1^*** | | **Early Globular *** | | **Late Globular** | | **Triangular** | | **Early /Mid Heart** | |
| --- | --- | --- | --- | --- | --- | --- | --- | --- | --- | --- |
|  | *Col-0* | *icr1* | *Col-0* | *icr1* | *Col-0* | *icr1* | *Col-0* | *icr1* | *Col-0* | *icr1* |
| **Non-stereotypic divisions in proembryo** | *0/105* | *0/98* |  |  |  |  |  |  |  |  |
| **Abnormal hypophysis specification** |  |  | *0/34* | *0/50* |  |  |  |  |  |  |
| **Abnormal specification of lens-shaped and large lower cells** |  |  |  |  | *0/56* | *0/38* |  |  |  |  |
| **Periclinal divisions in suspensor** | *0/105* | *0/98* | *0/34* | *0/50* | *0/56* | *0/38* | *0/35* | *2/27*  *(7%)* |  |  |
| **Periclinal/irregular divisions in protoderm** |  |  |  |  | *0/56* | *0/38* | *0/35* | *5/27*  *(18%)* | *0/19* | *5/33*  *(15%)* |
| **Extra-divisions in QC** (3 cells at early/mid heart stages and 3-5 cells at cotyledon stage) |  |  |  |  |  |  | *0/35* | *0/27* | *0/19* | *9/33*  *(27%)* |
| **Irregular division**  **planes in columella initials** |  |  |  |  |  |  | *0/35* | *8/27*  *(30%)* | *0/19* | *31/33*  *(94%)* |

Table S1: **The frequency of *icr1^-/-^*embryos exhibiting patterning defects at indicated developmental stages.**

***** Embryos with strong basal defects were excluded from analysis**.**

**^1^** Embryos were analyzed at 1-cell, 2-cells, 4-cells, 8-cells (octant) and 16-cells (dermatogen) stages.
